# CRISPR-mediated promoter editing of a *cis*-regulatory element of *OsNAS2* increases Zn uptake/translocation and plant yield in rice

**DOI:** 10.3389/fgeed.2023.1308228

**Published:** 2024-01-23

**Authors:** Yvonne Ludwig, Conrado Dueñas, Erwin Arcillas, Reena Jesusa Macalalad-Cabral, Ajay Kohli, Russell Reinke, Inez H. Slamet-Loedin

**Affiliations:** ^1^ International Rice Research Institute, Rice Genetic Design and Validation Unit, Rice Breeding Innovations, Los Baños, Philippines; ^2^ Department of Biology and Biotechnology “L. Spallanzani”, University of Pavia, Pavia, Italy

**Keywords:** biofortification, rice, CRISPR-Cas9, Zn, Fe, *OsNAS2* promoter, promoter editing

## Abstract

Developing nutritious rice with a higher yield is one approach to alleviating the problem of micronutrient deficiency in developing countries, especially human malnutrition involving zinc and iron (Fe) deficiency, and achieving better adoption. The transport of micronutrients such as Fe and Zn is mainly regulated via the nicotianamine synthase (*OsNAS*) gene family, whereas yield is a complex trait that involves multiple loci. Genome editing via CRISPR (clustered regularly interspaced short palindromic repeat)-Cas9, focusing on the *OsNAS2* promoter, particularly the deletion of the *cis*-regulatory element *ARR1AT* at position −933, was conducted for an enhanced accumulation of Zn in the grain and per plant. The results showed that our promoter editing increased Zn concentration per plant. Evidence also showed that an improved spikelet number per main panicle led to increased grain per plant. The traits were inherited in “transgene-free” and homozygous plant progenies. Further investigation needs to be conducted to validate trait performance under field conditions and elucidate the cause of the spikelet increase.

## 1 Introduction

A simple meal can satisfy hunger, but only an enriched, nutritious meal can help overcome “hidden hunger.” Nutrient deficiency is more common in Africa, South Asia, and Latin America, affecting approximately three billion people worldwide (Kumar et al., 2019). Widespread malnutrition deficiencies are iron-deficiency anemia, zinc deficiency, and vitamin A deficiency, which cause severe and long-lasting effects on the human body, such as stunting intellectual and physical growth or diminishing immune system response (Majumder et al., 2019). During pregnancy, a diet with malnutrition increases the risk of giving birth to a low-birth-weight baby, thus affecting fetal growth and even risking the infant’s survival (Kumar et al., 2019).

Micronutrients play various key roles in plant metabolism and homeostasis. One essential micronutrient is zinc (Zn), which is involved in numerous functions or biochemical processes in plants, such as acting as a cofactor, synthesizing nucleotides, and activating enzymes or plant growth hormones (e.g., auxin). Similarly, Zn is involved in plant growth and metabolic processes, such as gene expression and the synthesis of proteins, lipids, and nucleic acids (Zaman et al., 2018). Iron (Fe) is also a vital micronutrient that plays a significant role in plant physiological and biochemical pathways. It is a component of various enzymes, such as cytochromes, and is involved in the maintenance of chloroplast structure and function, as well as chlorophyll synthesis and respiration (Rout and Sahoo, 2015). However, most staple foods, such as rice, maize, and wheat, are inherently low in micronutrient content and cannot meet the daily requirements. Therefore, a growing challenge is to increase the nutritional value of crops, especially rice. Approximately 3.5 billion people consume rice; however, because of the extensive grain processing (polishing, steaming, and parboiling), rice loses a considerable quantity of micronutrients before consumption (Prom-u-thai et al., 2020). The nutritional quality of polished rice needs to be increased for Zn and Fe by 12 ppm and 11 ppm, respectively, from their baselines (2 ppm for Fe and 16 ppm for Zn) to be able to meet the 30% estimated average requirement (EAR) (Van Der Straeten et al., 2020). Because of constraints on the limited natural rice variation with increased Zn and Fe content in conventional breeding, genetic engineering is a helpful tool for generating rice plants with elevated micronutrient content. Transgenic approaches mainly target the key genes for the translocation, storage, and uptake of Fe and Zn. Different strategies, such as overexpression of single or multiple genes or silencing, successfully increased grain Fe and Zn concentrations.

Studies have reported increased micronutrient concentrations by overexpressing the *OsNAS2* gene in rice (Johnson et al., 2011) or by combining genes (*OsNAS2* and *HvNAAT*), which revealed an elevated Fe concentration of up to 55 μg/g (Diaz-Benito et al., 2018). Trijatmiko et al. (2016) reported encouraging results with up to 15 μg/g Fe and 45.7 μg/g Zn in polished rice from transgenic plants grown under field conditions. Wu et al. (2019) created transgenic rice lines expressing *AtNRAMP3*, *AtNAS1*, and *PvFer* cassettes that reached 48.18 μg/g Zn and 13.65 μg/g Fe in brown rice. The majority of studies have reported increased Zn/Fe concentrations through the insertion of genetic material from the same or different species into the rice genome to enhance their uptake, translocation, or storage (Ludwig and Slamet-Loedin, 2019). Future strategies for rice biofortification will use the CRISPR system, which has become a standard tool in gene editing. One important aspect is the possibility of fast-generating “transgene-free” plants with the desired trait improvement.

Many agricultural traits are related to yield indirectly, such as plant height, panicle length, seed length, and growth period, or directly, such as spikelet number per plant, panicle number per plant, and 1000-grain weight (Li et al., 2019). Using CRISPR-Cas9, several attempts have been made to improve rice yield. The target genes were *Gn1a* (grain number 1a), *DEP1* (dense and erect panicle 1), *GS3* (grain size 3), and *IPA1* (ideal plant architecture 1). T_2_ generation mutation analysis revealed enhanced grain number, dense erect panicles, and larger grain size (Li et al., 2016). Other studies involved *OsGS3*, *OsGW2*, and *OsGn1a* in a multiplex genome-editing approach, resulting in a triplet mutant and 30%–68% more yield per panicle (Zhou et al., 2019). In addition, phytohormones are known to be related to yield, such as abscisic acid (ABA) and cytokinin (CK), which are involved in plant growth and development and stress response. The ABA receptors pyrabactin resistance 1-like (PYL1), PYL4, and PYL6 were targeted using a genome-editing approach, resulting in up to 31% more grain than that in the wild-type counterparts under field conditions (Miao et al., 2018). Edited plants with mutated *OsLOG5*, targeting the rice cytokinin-activation enzyme-like gene, display seed traits with significantly higher numbers (increased seed setting rate, total grain number, grain number per panicle, and 1000-grain weight) than the control plants (Wang et al., 2020).

*OsNAS2* and other Zn transporter and acquisition genes are known to be regulated via the type-B OsRR-mediated signaling pathway (Gao et al., 2019). The possible disruption of the binding site, known as the *ARR1AT* motif, of OsRR could likely decrease the inhibitory effect on Zn uptake and/or translocation and lead to increased Zn concentration within the crop plant and/or grain. The Zn concentration is correlated with cytokinin concentration (Gao et al., 2019): higher Zn per plant could lead to an increase in cytokinin concentration and, subsequently, an improvement in yield-related traits such as panicle branching and seed development.

This research studied the *OsNAS2* promoter sequence modification introduced using the genome-editing tool CRISPR in the elite rice variety IR64. Three IR64-CRISPR lines targeting an *ARR1AT* motif were analyzed over several generations, and candidate plants were selected based on increased Zn and Fe concentrations in rice grain, the absence of the nuclease and biomarker, and a possible homozygous mutation. Consequently, the T_3_ “transgene-free” and homozygous plants were analyzed for yield-related traits and micronutrient concentrations.

## 2 Materials and methods

### 2.1 Promoter *cis*-regulatory element analysis

The promoter was analyzed for locating CREs using New PLACE (https://www.dna.affrc.go.jp/PLACE/?action=newplace, Higo et al., 1999). The region 1,000 bp upstream of the translational *OsNAS2* start was analyzed, and all *ARR1ATs* and *GTGANTG10* CREs were identified to select the possible on-target site. Subsequently, the edited on-target region was analyzed to identify the missing *ARR1AT* motif in the candidate plants.

### 2.2 Designing of single-guide RNA, vector construct, and indica rice transformation

The single-guide RNA (sgRNA) sequences targeting the *OsNAS2* promoter were identified using CRISPR-P (http://crispr.hzau.edu.cn/CRISPR/, Version 1.0, Lei et al., 2014). Oryza indica (ASM465v1) was used as the reference genome, and the reference sequence was obtained from the Gramene *Oryza sativa* indica group (ASM465v1, www.gramene.org). sgRNA was selected based on the on-target score and off-target sites (Table 1). A compact CRISPR vector system was used to generate genetically modified plants. The cloning and *Agrobacterium* transformation were performed as described in Ludwig and Slamet-Loedin (2020) to develop a CRISPR construct (Supplementary Figure S1), namely, IRS1421 (sgRNA11).

**TABLE 1:** sgRNA11 summary based on CRISPR-P.

Name	Sequence	PAM	Start location	On-target score	No. of off-target sites	Off-target locus	Off-target gene	Off-target region
sgRNA11	GGC​GTG​TAT​AAA​TCT​GTG​AA	AGG	3: 12,058,387	98	10	1:+17,442,667	BGIOSGA003557	CDS
						12:+13,677,335		Intergenic
						10: 16,697,590		Intergenic
						6:+23,855,906		Intergenic
						1:+45,318,422		Intergenic
						7: 26,838,590		Intergenic
						8: 1,3,561,876	NCRNA_44687	Exon
						11: 6005200	BGIOSGA035036	CDS
						11:+14,675,123	BGIOSGA035314	CDS
						11:+2,108,302		Intergenic

### 2.3 Plant growth

After being generated in the tissue culture facility, the T_0_ seedlings were transferred into the CL4 greenhouse bays at the International Rice Research Institute (IRRI). The genome-edited plants were planted singly into pots and grown at 28°C ± 7°C day temperature and 23°C ± 4°C night temperature. For the analysis of T_1_, T_2_, and T_3_ plant generations, the dormancy of 25–50 rice seeds per plant line was broken at 45°C. The seeds were seeded and, after germination, sown on soil-filled trays. After 18 days, the rice seedlings were transplanted Into the wet bed or wet bed and pot (T_2_ generation) of the transgenic screenhouse at IRRI.

### 2.4 Nuclease and biomarker screening

In the T_0_ and following generations, the presence of the Cas9 nuclease and the biomarker hygromycin phosphotransferase (HPH) was determined via a PCR approach. The KAPA3G Plant PCR Kit (Sigma-Aldrich) or the Phire Plant Direct PCR Kit (Thermo Fisher) were used for amplification with specifically designed Cas9 primers, generating a 2,132-bp amplicon in the presence of the nuclease. For the identification of HPH, a 554-bp PCR amplicon was detected. The same primers were used to analyze the T_1_, T_2_, and T_3_ rice plant generations for “transgene-free” plants.

### 2.5 T7 endonuclease assay

The sgRNA11 on-target site was amplified in a 15 µL PCR mixture using specific primers (Supplementary Table S1), and the PCR products (5 µL) were analyzed by 1% agarose gel electrophoresis. Only plants with strong single-amplicon signals with the expected size were used for the T7E1 assay. A measure of 4–5 µl of the PCR product was mixed with NEBuffer 2 and incubated at 95°C for 10 min, 85°C for 5 min, and 25°C for 5 min and stored at 10°C. Afterward, 0.5 U T7 endonuclease 1 was added, and the mixture was incubated at 37°C for 1 h. Consequently, the reaction products were analyzed via gel electrophoresis. Because of the inability to differenciate between homozygous and wild type plants, all single amplicon samples were reanazlyed as follow to determine the zygosity: the genome-edited PCR amplicon was mixed with the IR64 wild-type PCR amplicon (1:1) and analyzed as described before.

### 2.6 Seed processing for X-ray fluorescence and/or inductively coupled plasma-optical emission spectrometry

Paddy rice was manually dissected to remove the hull; it was broken open using forceps to extract the brown rice seed. A measure of 3–4 g of brown rice (∼150 seeds) was prepared for X-ray fluorescence (XRF) measurement. Each sample was measured twice.

For inductively coupled plasma-optical emission spectrometry (ICP-OES), the samples were polished for 55 s using a Kett Pearlest Grain Polisher. The rice seeds were then visually examined for uncleanliness and corrected if necessary.

### 2.7 Amplification of sgRNA on-target site and sequencing

Specific primers (Supplementary Table S1) were designed to amplify the target site of sgRNA11. An 898-bp PCR amplicon containing the target region of genome-edited rice lines was either directly sent for sequencing or cloned into the sequencing vector pGEM-T Easy or TOPO-TA for further analysis.

### 2.8 T_0_ plant analysis

The leaf samples of the T_0_-generated IR64 seedlings were taken, and genomic DNA was extracted using a modified CTAB method. All regenerated plants were screened for the presence of the Cas9 nuclease and biomarker HPH via a PCR approach to detect the existence of T-DNA in the T_0_ generation. Plants without T-DNA insertion were discarded. The remaining plants were screened via the T7E1 assay to determine mutations within the region of interest. Subsequently, the promoter sites were amplified with specific primers (Supplementary Table S1) and sequenced by Sanger sequencing. The sequencing files were analyzed using Synthego ICE (https://ice.synthego.com/#/, Conant et al., 2022) to evaluate and identify variations in the amplicons. The seeds were harvested and processed. Brown rice seeds were evaluated for micronutrient content via XRF to detect plants with higher Zn and Fe concentrations than those of the control plants (tissue culture control named TC).

### 2.9 T_1_/T_2_/T_3_ plant analysis

Three candidate events (IR64-IRS1421-001, IR64-IRS1421-026, and IR64-IRS1421-036) were selected from the T_0_ generation for further analysis. The subsequent generations were analyzed to confirm the germline-editing search for “transgene-free” plants and higher Zn and Fe concentrations. All plants were analyzed via PCR for the presence of the Cas9 nuclease and the biomarker HPH to identify “transgene-free” rice plants. Brown rice (T_3_ and T_4_ seeds) and polished rice (T_2_ seeds) were submitted for XRF and ICP-OES analyses, respectively. Furthermore, based on the screening results and micronutrient concentrations, “transgene-free” or candidate plants (nuclease- or biomarker-free) were evaluated for sequence variation in the sgRNA11 target sites via the T7E1 assay. Consequently, PCR productor Plasmid DNA was sent (T_2_ and T_3_ plants) or plasmid DNA (T_1_ plants) were sent for Sanger sequencing. Through Synthego ICE, the sequencing files were analyzed to identify variations within the target sequence. Agronomic characteristics of the T_3_ plant generation, such as plant yield, plant height, tiller number, and panicle characteristics, were analyzed compared to wild-type plants with edited “transgene-free” generations.

### 2.10 Panicle analysis

The main panicle was harvested and then spread on a white background. Pictures were taken using a Nikon D5200 camera (lens: Nikon DX AF-S NIKKOR 18–105 mm 1:3.5-56G ED). The panicle analysis was performed using the widely used open-source image analysis software ImageJ (Schneider et al., 2012). Different panicle traits were analyzed: rachis length, primary branch number (PBN), secondary branch number (SBN), primary branch average length (PBL), secondary branch average length (SBL), secondary branches per primary branch (SBperPB), and spikelet number.

### 2.11 Off-target analysis

Off-target analysis was carried out using PCR and a T7 endonuclease assay. Specific primers (Supplementary Table S2) were used to amplify the identified 10 off-target sites of sgRNA11 using either the KAPA3G Plant PCR Kit (Sigma-Aldrich) or the Phire Plant Direct PCR Kit (Thermo Fisher). A measure of 5 µl of the PCR product was analyzed by 1% agarose gel electrophoresis. The T7E1 assay was then performed on the samples with the expected band size. A measure of 3 µl of the PCR product was mixed with NEBuffer 2, water, and 3 µL of IR64-wild-type PCR amplicon and incubated at 95°C for 10 min, 85°C for 5 min, and 25°C for 5 min and stored at 10°C. T7 endonuclease 1 (0.5 U) was added, and the mixture was incubated at 37°C. Lastly, digestion products were visualized via 1.2% agarose gel electrophoresis.

### 2.12 RT-PCR analysis

Leaf samples were harvested in the late-flowering stage, and total RNA was extracted utilizing the RNeasy Mini Kit with the RNase-Free DNase Set (QIAGEN), according to the manufacturer’s instructions. Subsequently, reverse transcription was performed using the Transcriptor First Strand cDNA Synthesis Kit (Roche). Quantitative PCR was then conducted in 20 μL reaction volumes using the SYBR Select Master Mix (Applied Biosystems) on a 7500 Fast Real-Time PCR system (Applied Biosystems). The primer efficiency of each oligonucleotide was calculated using the following dilution series: 1, 1/2, 1/4, 1/8, 1/16, and 1/32. Using the Pfaffl method, the efficiency-corrected relative gene expression was calculated with reference to the housekeeping gene *OsTPI*. For each edited rice line and wild-type, three and nine biological replicates were analyzed, respectively. Three technical replicates were measured for each sample.

## 3 Results

### 3.1 Promoter *cis*-regulatory element analysis

**FIGURE 1:**
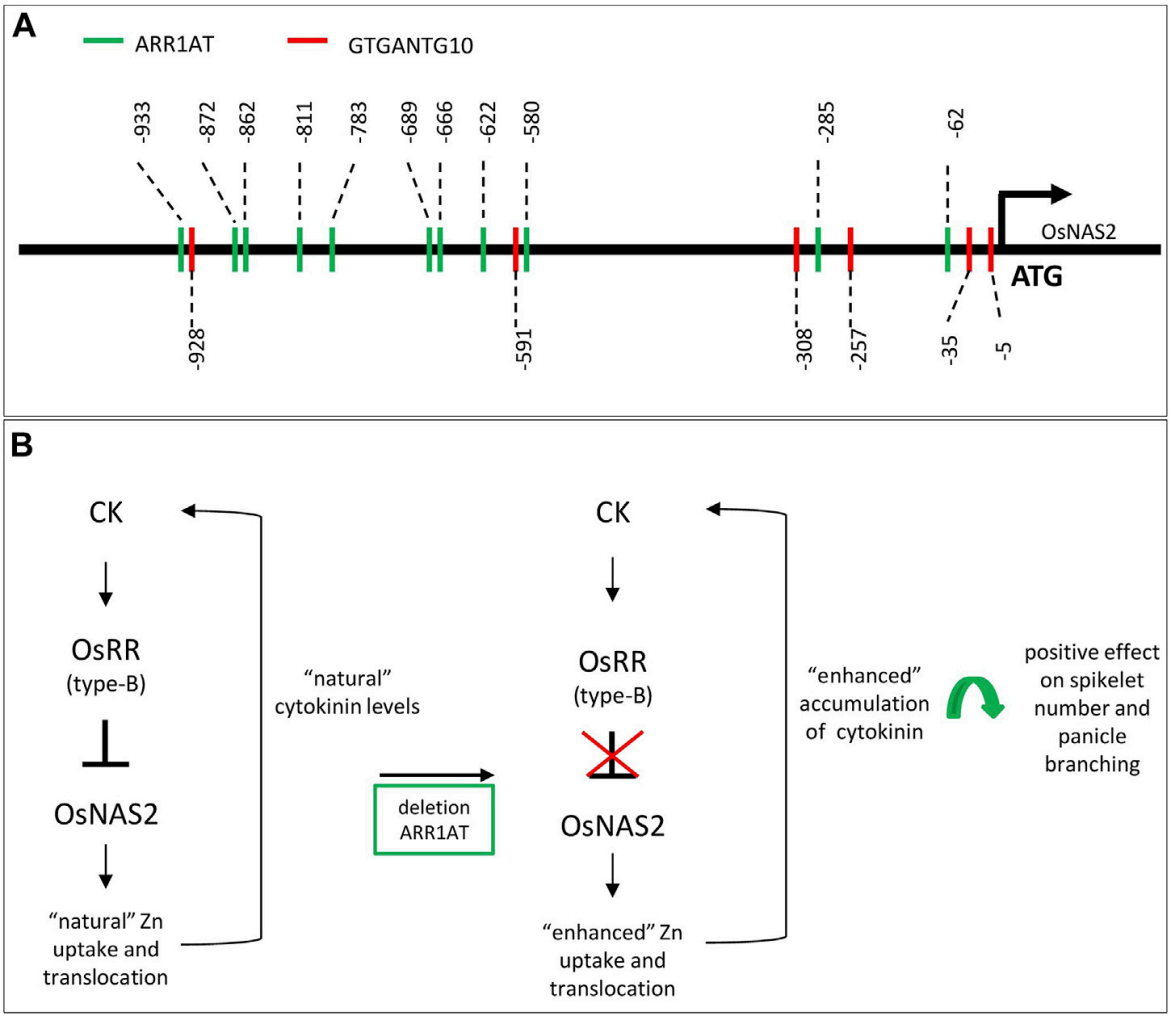
Promoter structure of *OsNAS2* in elite rice IR64 and the theorized mechanism for enhanced Zn concentration in rice grain: **(A)** identification of CRE *ARR1AT* (in green) and *GTGANTG10* (in red) within the 1-kb promoter region. **(B)** Hypothetical network of the positive effect on seed number and panicle branching after disruption of the *ARR1AT* motif in the *OsNAS2* promoter region. CK, cytokinin; OsRR, *Oryza sativa* spp. response regulators.

A 1-kb-upstream *OsNAS2* promoter analysis revealed a total of 11 *ARR1AT* (site #S000454) and 6 *GTGANTG10* (site #000378) motifs (Figure 1A), in which the target *ARR1AT* motif is at position −933 and was deleted through the genome editing approach. At the same time, the CRE *GTGANTG10* motif at position −928 was disrupted as well. The *ARR1AT* motif is an ARR1-binding element identified in *Arabidopsis thaliana* as a response regulator (Sakai et al., 2000) and is linked to cytokinin regulation (Melton et al., 2019). *GTGANTG10*, with its GTGA motif, is also found in the tobacco (*Nicotiana tabacum*) promoter of the late pollen gene g10 and is a homolog of the tomato gene lat56, regulating sperm cell-specific gene expression (Rogers et al., 2001).

### 3.2 Analysis of elite rice variety IR64 plants edited by CRISPR-Cas9

#### 3.2.1 T_0_ generation: transformation efficiency evaluation, CRISPR-Cas9-induced sequence variation, and Zn/Fe concentrations in candidate events

A total of 101 IR64-IRS1421 T_0_ plants were generated via *Agrobacterium tumefaciens*-mediated transformation. All generated T_0_ plants were screened for the Cas9 nuclease located close to the right border (RB) and the biomarker HPH located near the left border (LB) by PCR using sequence-specific oligonucleotides (Supplementary Table S1) to confirm the full T-DNA insertion. A transformation efficiency of 89.11% was detected, and overall, 92 plants (91.09% mutation rate) were identified with a sequence modification via the T7E1 assay (Table 2). Taking into consideration the T7E1 screening as well as the Cas9/HPH test, a total of 67 T_0_ plants were sequenced, and 29 different alleles were identified (Supplementary Figure S2).

**TABLE 2:** Screening results throughout the generations (T_0_–T_3_).

Event ID	No. of plants	Cas9^+^	HPH^+^	T7E1^+^	Homozygous	Transgene free	Mutation rate (%)	Transformation efficiency (%)
***T***_***0***_ ***generation*** (***total events***)								
IR64-IRS1421	101	90	99	92	0	0	91.09	89.11
***T***_***1***_ ***generation*** (***candidates only***)								
IR64-IRS1421-001	20	9	12	11	0	2	55	
IR64-IRS1421-026	20	13	14	4	1	1	20	
IR64-IRS1421-036	19	12	14	10	0	3	52.63	
***T***_***2***_ ***generation*** (***candidates only***)								
IR64-IRS1421-001-009	20	4	2	13	0	12	65	
IR64-IRS1421-026-007	18	9	11	18	0	4	100	
IR64-IRS1421-036-006	19	10	17	17	6	2	89.47	
***T***_***3***_ ***generation*** (***candidates only***)								
IR64-IRS1421-001-009-004	24	0	7	19	8	17	79.17	
IR64-IRS1421-001-009-005	24	0	7	20	13	17	83.33	
IR64-IRS1421-026-007-001	24	0	6	24	17	18	100	
IR64-IRS1421-026-007-002	24	0	2	24	23	22	100	
IR64-IRS1421-026-007-003	24	0	3	24	24	21	100	
IR64-IRS1421-036-006-002	24	0	10	24	24	14	100	
IR64-IRS1421-036-006-008	24	2	9	24	10	15	100	
IR64-IRS1421-036-006-009	24	21	24	24	24	0	100	

**FIGURE 2:**
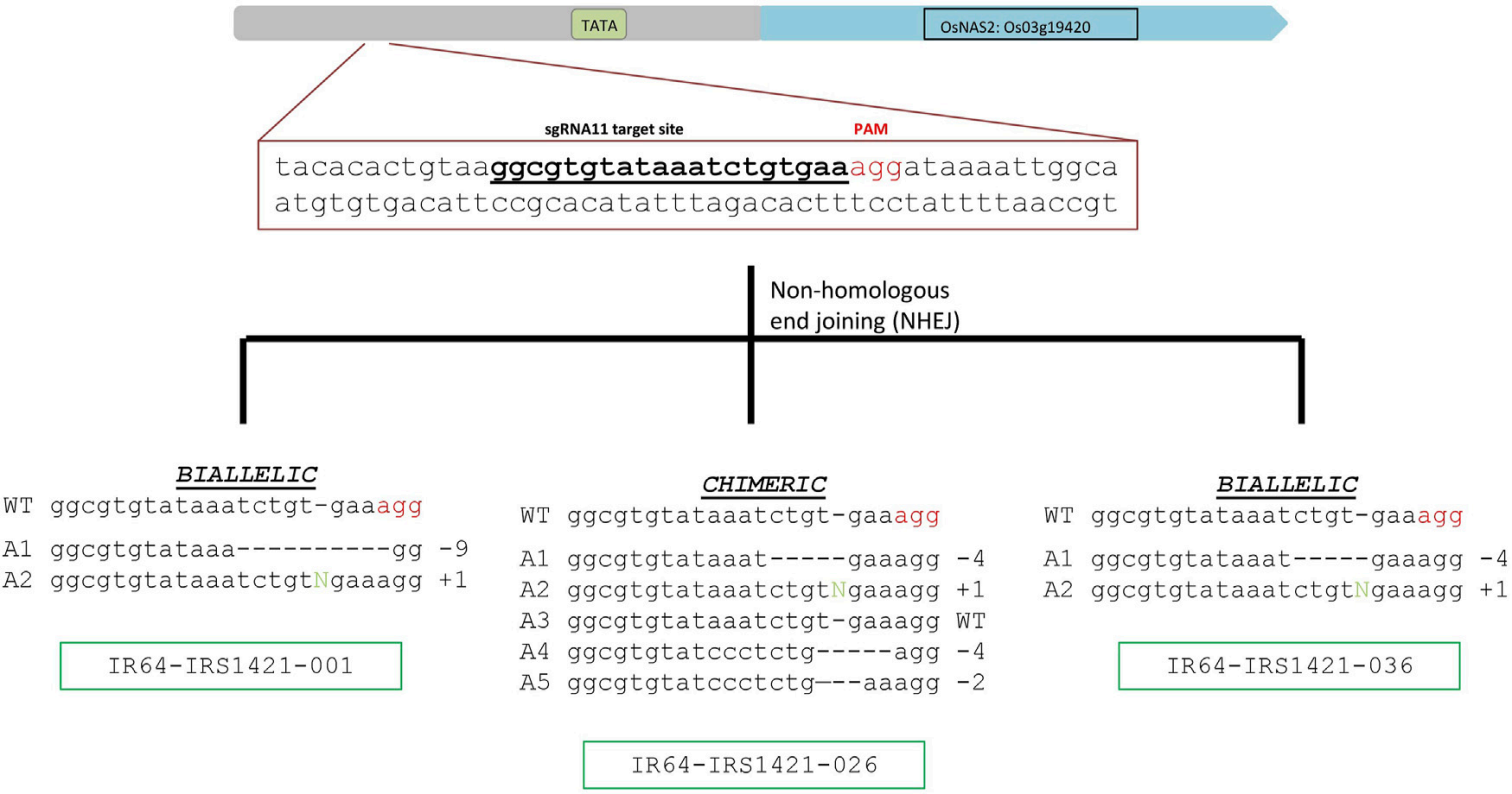
sgRNA target site and identified alleles in the selected T_0_ candidates: sgRNA11 target-site sequence and PAM (indicated in red letters), as well as the different alleles identified in the selected candidates. Two candidates are biallelic (IR64-IRS1421-001 and IR64-IRS1421-036), and IR64-IRS1421-026 is chimeric, with four different allele variations identified.

**FIGURE 3:**
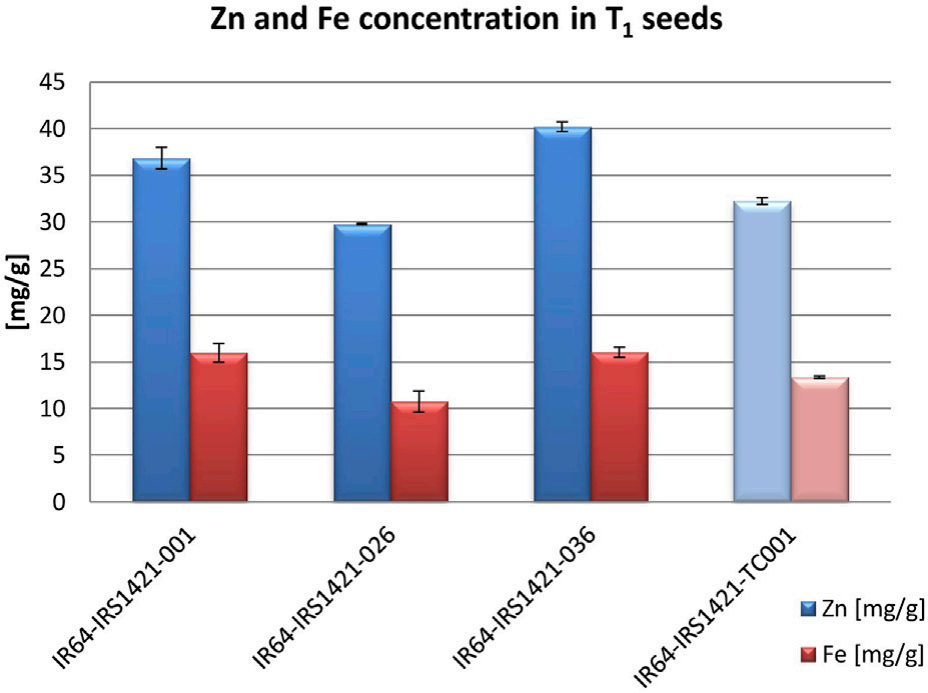
T_1_ seed Zn and Fe concentrations compared to the tissue culture control (TC): 150 brown rice seeds from each candidate and control plant were measured using XRF. Each sample was measured twice. The standard error indicates the difference in the measurements of each sample.

Based on different sequencing results, 31 events were selected and evaluated for Zn and Fe concentrations via XRF. Three candidate events (IR64-IRS1421-001, IR64-IRS1421-026, and IR64-IRS1421-036) were selected, displaying diverse characteristics in identified sequence variation (Figure 2) and increased Zn/Fe concentrations in brown rice (Figure 3).

#### 3.2.2 T_1_/T_2_ generation: screening and selection process to identify plant lines with increased Fe and Zn concentrations in rice grain

**FIGURE 4:**
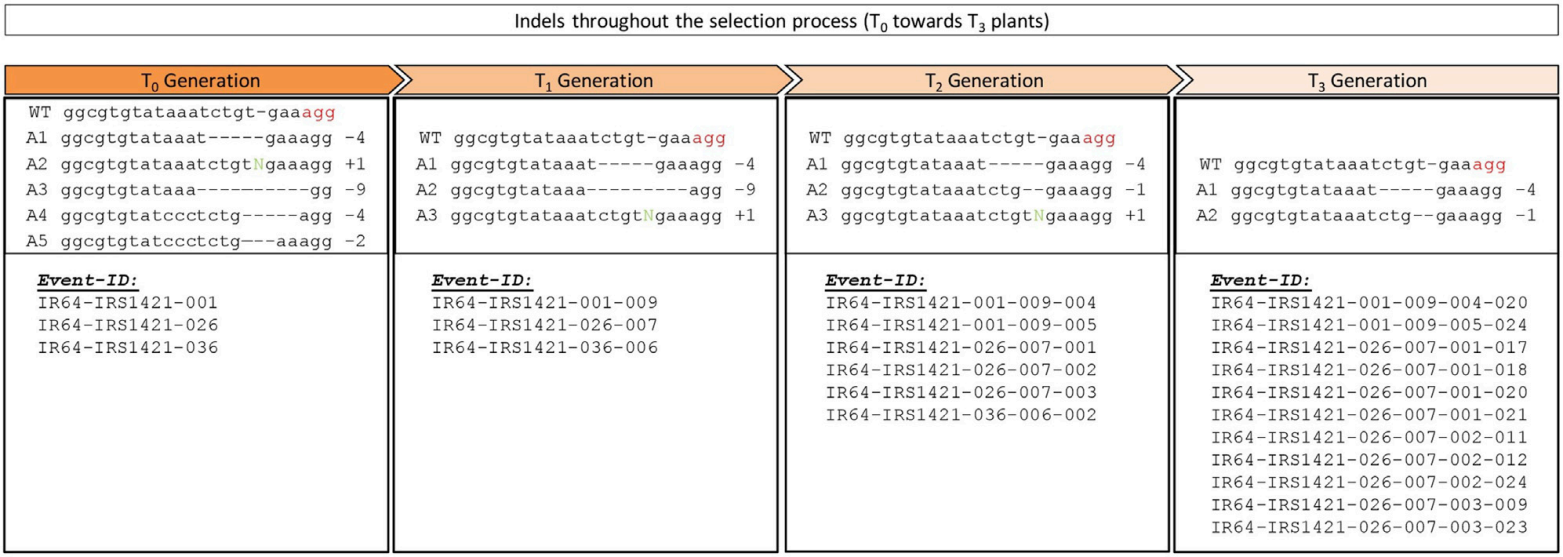
Allele variation throughout the selection process from T_0_ to T_3_: different alleles identified in each generation and their corresponding Event ID.

The genome-edited plants of the T_1_ and T_2_ generations were grown in the transgenic screenhouse at IRRI, and the leaf samples were taken 2–3 weeks after transplanting them into the wet bed (T_1_) or wet bed and pot (T_2_). Genomic DNA was extracted from young leaves and used for screening the presence and absence of the nuclease and biomarkers. IR64-IRS1421-001-T_1_ plant screening conferred two “transgene-free” plants (no Cas9 and HPH were detected) out of 20 plants. Most T_1_ plants still had the nuclease and/or biomarker presence (Table 2; Supplementary Figure S3). All 20 tested plants were analyzed via T7E1 and/or sequencing and were either still biallelic or had a wild-type allele. The IR64-IRS1421-036-T_1_ generation had three “transgene-free” plants, and the IR64-IRS1421-026-T_1_ generation displayed only one plant out of 19 or 20 plants, respectively. Similarly, for these two candidate lines, most plants thus far presented Cas9 and HPH amplicons (Table 2). Furthermore, the sequencing analysis and T7E1 screening showed biallelic or wild-type plants. Only IR64-IRS1421-026-007 was identified as a homozygote. Unfortunately, the plants still presented the Cas9 and HPH amplicons. After preliminary selection, 14 T_2_ rice seed samples were sent for ICP analysis (Supplementary Table S3). Considering the genotypic and phenotypic data, three candidate plants (IR64-IRS1421-001-009, IR64-IRS1421-026-007, and IR64-IRS1421-036-006) were chosen, one for each candidate event. The T_2_ plant generation was screened as described for the T_1_ plant generation. Only the IR64-IRS1421-001–009-T_2_ generation displayed four and two plants positive for Cas9 and HPH, respectively. Half or most of the total T_2_ plant generation still presented the nuclease and biomarker amplicon for the other two candidate lines (Table 2). An increased mutation rate (Table 2) was obtained in each generation by eliminating the heterozygous plants. Selection was carried out to generate “transgene-free” homozygous plants. The T_3_ seeds of eight candidate lines were sent for XRF analysis to determine the Zn and Fe concentrations in the brown rice (Supplementary Table S4). Selection for the following generation was first based on the Zn/Fe concentrations in the brown rice, followed by the presence and absence of Cas9 and HPH. For example, IR64-IRS1421-026-007 lines are “transgene-free” as opposed to IR64-IRS1421-001-009 and IR64-IRS1421-036-006 lines. Interestingly, the indels manifested within the germline (Figure 4) throughout the generations correspond to the increased Zn/Fe concentrations in the rice grain.

#### 3.2.3 T_3_ generation: identification of “transgene-free” plants with a homozygous mutant in the promoter target region and their respective Zn/Fe concentrations in T_4_ brown rice

**FIGURE 5:**
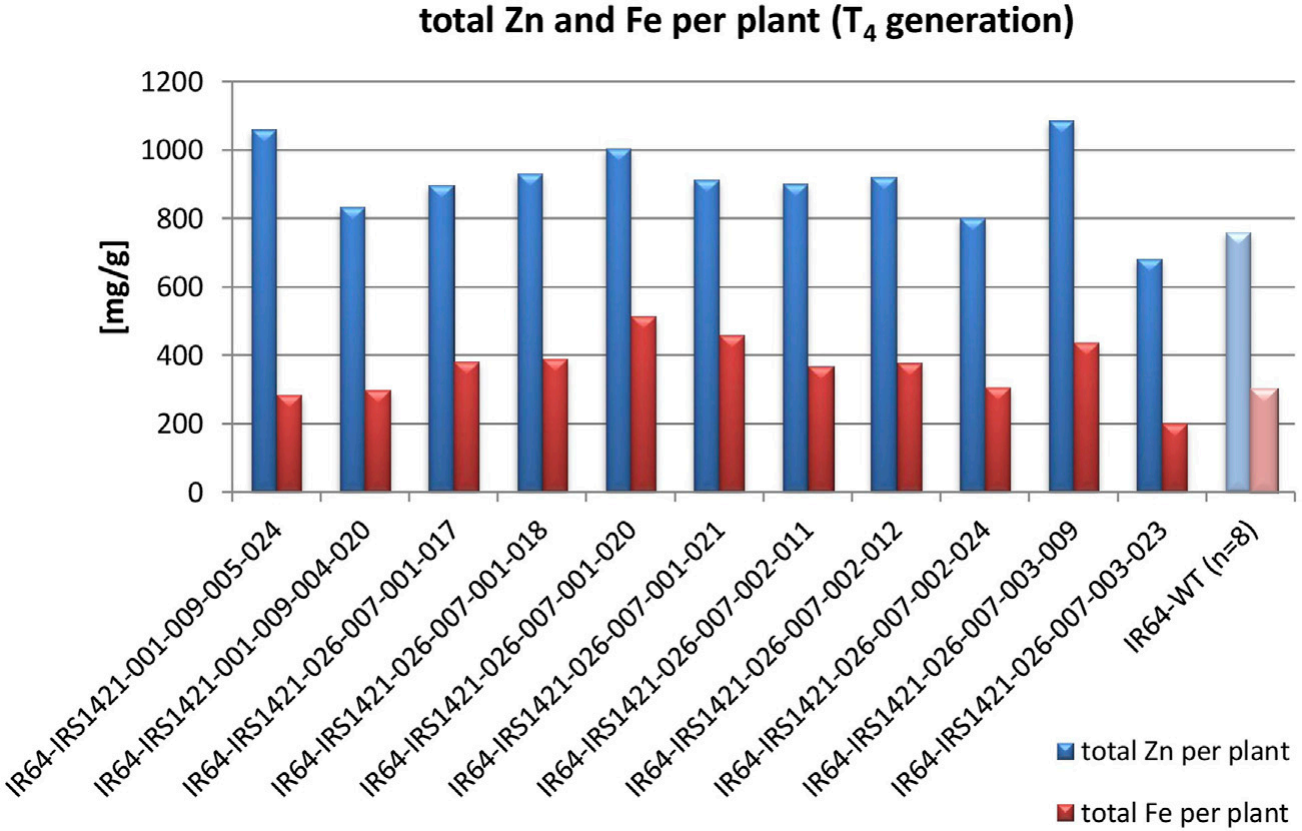
Estimated Zn and Fe concentrations per plant of selected T_4_ candidates compared to the IR64 wild-type control: Zn and Fe concentrations were measured via XRF, and the total Zn and Fe concentration per plant was calculated based on the total plant yield.

The majority of the plants in the T_3_ generation are Cas9-free or “transgene-free”, except for IR64-IRS1421-036-006-009, which has only three Cas9-free plants and all still display HPH (Supplementary Figure S3). However, the mutation rate within the T_3_ plant generation (192 plants in total) is in the range of 80%–100%, which is mainly homozygous (Table 2). The sequencing results of the homozygous plants display either a 4-bp or 1-bp deletion at the sgRNA11 on-target site (Figure 4). Furthermore, the Zn and Fe concentrations of T_4_ brown rice were detected via XRF. The estimated Zn concentration was calculated with respect to the total yield per plant to obtain an overview of the general Zn distribution per plant (Supplementary Figure S4). Taking these results into account, 11 “transgene-free” and homozygous rice plants were selected for further studies. The 4-bp-deletion plants displayed significantly higher grain number per plant, total grain weight per plant, flag leaf width, panicle length, filled grain number, and plant height but did not differ significantly in other phenotypic traits vis-à-vis the IR64 wild-type control (Table 3; Supplementary Table S5) and had a higher Zn concentration per plant than the wild type (Figure 5). Compared with the wild-type plant, the 1-bp deletion plants displayed significant differences in panicle count, 100-seed weight, and rachis length only (Supplementary Table S5). The types of mutations in these progenies correspond to the mutations in their parental lines (Figure 2).

**TABLE 3:** Agronomic data of selected T_3_ rice plants.

Event ID	del	Plant height (cm)	Tiller count	Panicle count	Flag-leaf length (cm)	Flag-leaf width (cm)	Panicle length (cm)	Filled grain number	Unfilled grain number	Total grain number per plant	Total grain weight per plant (g)	100-seed weight (g)	Fertility (%)	Fe (mg/g)^a^	Zn (mg/g)^a^
IR64-IRS1421-001-009-005-024	1 bp	114	23	23	56	1.7	32	1,839	1735	3,574	43.17	2.46	51.45	6.60	24.60
IR64-IRS1421-001-009-004-020		121	22	22	45	1.7	30.4	1,232	1,409	2,641	29.89	2.50	46.65	10.00	27.90
Average		117.5	22.5	22.5	50.5	1.7	31.2	1,535.5	1,572	3,107.5	36.53	2.48	49.05	8.30	26.25
IR64-IRS1421-026-007-001-017	4 bp	114	20	20	40	1.7	27	1,438	648	2,086	34.79	2.51	68.94	11.00	25.80
IR64-IRS1421-026-007-001-018		113	21	21	48	1.7	27	1,383	668	2,051	34.24	2.50	67.43	11.40	27.20
IR64-IRS1421-026-007-001-020		117	22	22	35	1.8	26	1,724	785	2,509	41.89	2.51	68.71	12.30	24.00
IR64-IRS1421-026-007-001-021		118	20	19	41	1.8	26	1,438	1,029	2,467	34.34	2.57	58.29	13.40	26.60
IR64-IRS1421-026-007-002-011		116	17	17	44	1.8	27	1,425	710	2,135	33.14	2.36	66.74	11.10	27.20
IR64-IRS1421-026-007-002-012		113	19	19	43	1.7	27	1,451	720	2,171	34.18	2.44	66.84	11.10	27.00
IR64-IRS1421-026-007-002-024		113	21	21	39	1.8	25	1,573	715	2,288	36.94	2.45	68.75	8.30	21.70
IR64-IRS1421-026-007-003-009		111	21	20	38	1.6	23.5	1,484	549	2,033	33.77	2.33	73.00	13.00	32.20
IR64-IRS1421-026-007-003-023		107	21	20	39	2	25	1,389	734	2,123	29.08	2.29	65.43	6.90	23.50
Average		113.56	20.22	19.89			40.78			1.77	728.67	2.44	67.12	10.94	26.13
IR64-WT (n = 8)		108.38	20.00	18.38	45.94	1.64	27.63	1,042.88	652.13	1,695	23.64	2.36	61.36	12.70	32.20

^a^ XRF measurement (T_4_ brown rice).

#### 3.2.4 Analysis of different panicle traits of the selected 11 T_3_ candidate plants

**FIGURE 6:**
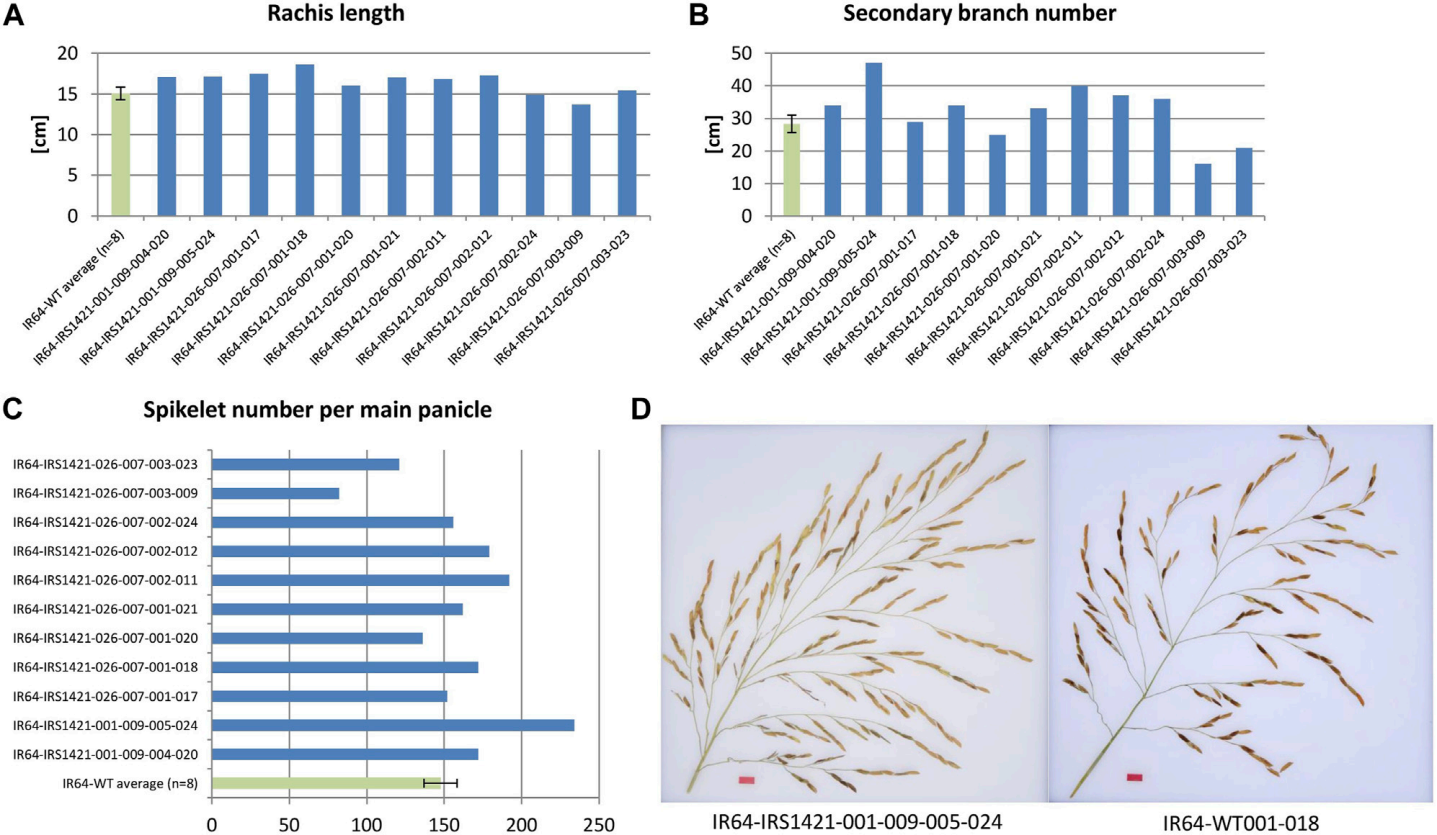
Presentation of measured panicle traits of T_3_ selected candidate plants. **(A, B)** Rachis length and secondary branch number (SBN) of the edited “transgene-free” plants compared to the IR64 wild-type plant (N = 8), respectively. **(C)** Spikelet number per main panicle of edited rice plants compared to the control (N = 8). **(D)** Example image of a main panicle of the edited plant IR64-IRS1421-001-009-005-024 and an IR64 wild-type plant.

Different panicle traits were analyzed: rachis length, primary branch average length (PBL), primary branch number (PBN), secondary branch average length (SBL), secondary branch number (SBN), secondary branches per primary branch (SBperPB), and spikelet number. Seven plants out of 11 displayed a longer rachis length than the wild-type control, and significant differences were observed for the 1-bp-deletion plants compared with the wild type. The majority of edited “transgene-free” plants showed a similar trend, but no significant differences in secondary branch traits (SBN and SBperPB) and spikelet number per main panicle were observed (Figures 6A–D). The remaining panicle characteristics did not differ from those of the wild-type plants (Supplementary Table S5; Supplementary Table S6).

#### 3.2.5 Off-target analysis via the T7 endonuclease assay

All 11 plants were tested for off-target cutting via the T7 endonuclease assay. The off-target region was amplified using specifically designed oligonucleotides (Supplementary Table S2). All 11 rice plants in all 10 off-targets tested negative except for off-target 6, for which IR64-IRS1421-001-009-005-024 and IR64-IRS1421-001-009-004-020 displayed an extra band (Table 4; Supplementary Figure S5A). This one off-target cutting site is located in an intergenic region in chromosome 7 and does not affect any nearby genes. Furthermore, all 110 samples were sent for sequencing to confirm the T7E1 assay results (Supplementary Figure S5B).

**TABLE 4:** Mutation detection in 10 off-target (OT) sites of sgRNA11 in T_3_ rice plants.

Event ID	Mutation									
	*OT1*	*OT2^a^*	*OT3^a^*	*OT4^a^*	*OT5^a^*	*OT6^a^*	*OT7*	*OT8*	*OT9*	*OT10^a^*
IR64-IRS1421-001-009-005-024	ND	ND	ND	ND	ND	D	ND	ND	ND	ND
IR64-IRS1421-001-009-004-020	ND	ND	ND	ND	ND	D	ND	ND	ND	ND
IR64-IRS1421-026-007-001-017	ND	ND	ND	ND	ND	ND	ND	ND	ND	ND
IR64-IRS1421-026-007-001-018	ND	ND	ND	ND	ND	ND	ND	ND	ND	ND
IR64-IRS1421-026-007-001-020	ND	ND	ND	ND	ND	ND	ND	ND	ND	ND
IR64-IRS1421-026-007-001-021	ND	ND	ND	ND	ND	ND	ND	ND	ND	ND
IR64-IRS1421-026-007-002-011	ND	ND	ND	ND	ND	ND	ND	ND	ND	ND
IR64-IRS1421-026-007-002-012	ND	ND	ND	ND	ND	ND	ND	ND	ND	ND
IR64-IRS1421-026-007-002-024	ND	ND	ND	ND	ND	ND	ND	ND	ND	ND
IR64-IRS1421-026-007-003-009	ND	ND	ND	ND	ND	ND	ND	ND	ND	ND
IR64-IRS1421-026-007-003-023	ND	ND	ND	ND	ND	ND	ND	ND	ND	ND

^a^ Intergenic region; ND, not detected; D, detected.

#### 3.2.6 RT-PCR analysis of T_4_ homozygous, “transgene-free” plants

Six high-performing edited rice lines were analyzed via RT-PCR, and the leaf samples at the late-flowering stage were used to measure *OsNAS2* expression. Significantly higher expression of *OsNAS2* compared to the wild-type control was measured for IR64-IRS1421-001-009-005-024 only. The other five selected rice lines displayed a similar trend (Supplementary Figure S7).

## 4 Discussion

The overall high sgRNA11 efficacy observed in this study suggests a good selection process based on the criteria at that time since the CRISPR-Cas9-based sequence mutation mainly depends on the specific cleavage and effectiveness of sgRNA. The unique nucleotide sequences (8–12 nt) at the 3′-end of sgRNA and the total length and nucleotide composition affect the on- and off-target efficiency (Uniyal et al., 2019). Furthermore, the secondary structure plays a significant role in the efficiency of the CRISPR-Cas9 system: weaker guide sequence structures are more efficient than stronger guide sequence structures, taking into account the CG content range (30%–80%) and base-pairing scores (Jensen et al., 2017; Javaid and Choi, 2021). In addition, a short T-rich sequence (TTTT) at the 3′-end of sgRNA should be avoided; this motif blocks the efficiency of the genome-editing CRISPR-Cas9 system (Graf et al., 2019).

The T7 endonuclease I assay was used to validate the CRISPR-Cas9 edits, which is inexpensive, simple to use, and more sensitive for detecting mutants than the Surveyor assay (Vouillot et al., 2015). Nonetheless, assay performance can be influenced by different aspects such as length and base-pair mismatch identity, flanking sequence, secondary structure of the target, and mutant sequence abundance (Sadowski, 1971; Dickie et al., 1987; Mashal et al., 1995). Unfortunately, the T7E1 assay is not able to detect homozygous mutations. To make it capable of identifying the homozygous plants, the PCR amplicon of the on-target site of wild-type and genome-edited plants was mixed (1:1). Already, earlier studies demonstrated that T7E1 had a maximum efficiency in a DNA pool containing only 5%–30% mutated DNA (Vouillot et al., 2015), which is sufficient for indel analysis.

Engineering higher micronutrient content (such as Zn) is a challenging task in staple crops because of the involvement of diverse processes, from uptake by the plant root to translocation and accumulation in the grain or edible plant parts (Gao et al., 2019). The leading work for biofortification is the overexpression of different genes or gene combinations involved in uptake, translocation, or storage acquired from the same or other species (Ludwig and Slamet-Loedin, 2019). Similar challenges are known for improving yield in crops since grain yield is a complex trait controlled by multiple loci. This study initially focused on developing rice with enhanced micronutrient content; however, editing of a single or a few base pairs in the promoter of a target gene through the CRISPR-Cas9 genome-editing tool shows an increase in Zn concentration of the grain in T_4_ seeds and also a potential increase in yield-related traits per plant. The *OsNAS2* promoter was targeted because of the significant role of the *OsNAS2* gene in the uptake and translocation of Zn/Fe in the rice plant. Evidence shows that minor editing happened at the *cis*-regulatory element *ARR1AT*. The CRE *ARR1AT* is known to be the binding element of ARR1 found in *A. thaliana* (Sakai et al., 2000). Throughout the selection process, rice plants with enhanced Zn and/or Fe concentrations were selected and analyzed for the presence of biomarkers, nucleases, presence (T_0_)/absence (T_1_ ongoing), and sequence variations. The selected high-Zn and high-plant-yield-edited plants showed either a 4-bp or 1-bp deletion at the *ARR1AT* motif at position −933 and a possible correlation with the detected increased micronutrient concentrations. A recent study demonstrated that cytokinin plays a key role in controlling Zn in plants and in involving type-B OsRR as a regulator for *OsNAS* genes (Figure 1B) (Gao et al., 2019). One single cytokinin response element has been recognized, which is a type-B response regulator linked to the core sequence 5′-GAT (T/C)-3′ in *Arabidopsis* (Brenner et al., 2012). Analysis of the 1-kb promoter region upstream of the translational start of *OsNAS2* displayed 11 core sequence 5′-GAT (T/C)-3′ motifs (Figures A1–A). The Zn transporter and acquisition genes such as *OsNAS2* are regulated via the type-B OsRR-mediated signaling pathway (Gao et al., 2019). We hypothesize that by destroying one of the possible binding sites (*ARR1AT* motif) of the OsRR regulators, the inhibitory effect was minimized or stopped, and a desirable enhanced Zn uptake or translocation could occur (Figures A1–B). Few *in planta* studies have described loss-of-function mutations, such as mutations within the core sequence of CRE in the ARR6 promoter that strongly decrease the responsiveness to cytokinin in *Arabidopsis* (Ramireddy, 2009). In the MYB family, 11 type-B ARRs containing this N-terminal CRE are thought to be negatively regulated (Muthamilarasan et al., 2014). Moreover, the motif deletion led to a constitutively active protein Iform in *Arabidopsis* (Gao et al., 2019). In rice, it was demonstrated that cytokinin activated the promoter of *OsNSHB2* (non-symbiotic hemoglobin); deletions or mutations of the predicted ARR1-binding CRE in the promoter confirmed its function as a cytokinin response element. The motif deletion drastically decreased the inducibility of the promoter cytokinin (Ross et al., 2004). Gao et al. (2019) described the inhibitory effect of cytokinin on the expression of Zn acquisition and translocation genes (*OsNAS*, *OsZIP*, and *OsHMAs*) in rice. Moreover, studies have shown that cytokinin-deficient plants exhibit an elevated Zn concentration, and that Zn uptake is regulated by cytokinin in a dose-dependent manner (Gao et al., 2019). Accumulating data demonstrate that cytokinin plays an important role in responding to nutrient status (Sakakibara et al., 2006; Ramireddy et al., 2014; Gao et al., 2019) with its wide repression of nutrient transport (Sakakibara et al., 2006). Furthermore, it is known that cytokinin is involved in diverse physiological and developmental processes of the plant, such as shoot and root development, leaf expansion, vascular differentiation, senescence, and spikelet number per panicle (Deveshwar et al., 2020; Prasad, 2022). In addition, Gao et al. (2019) suggested that cytokinin concentration is correlated with Zn availability and that a possible spatial control of cytokinin metabolism could be exploited for crop nutritional improvement. Furthermore, cytokinin concentration affects SAM size and activity in plants and, subsequently, plant height, tiller number, and panicle branches (Du et al., 2017). The association of cytokinin with agronomic traits, particularly spikelet number, could be observed in the edited “transgene-free” plants.

Although Zn concentration still varies in T_4_ seeds, indications are given that an increased Zn concentration can be achieved by disrupting the *ARR1AT* motif. In addition, evidence shows that yield-related traits, such as spikelet number, increased in most selected plants. Much research is required to further analyze the following generation of currently achieved “transgene-free” homozygous plants. Despite that, it has been shown that the same genotype (gene, QTL, etc.) can have a diverse or even opposite effect in a different genetic background (Shen et al., 2018). A feasible next step could be backcrossing to eliminate possible somaclonal mutations to clean up the genetic background.

In this study, we hypothesize that the disruption of the CRE *ARR1AT* motif at position −933 in the *OsNAS* promoter can enhance Zn concentration in the rice plant and have a positive effect on yield-related traits such as spikelet number and panicle branching. The estimated total Zn concentration is still diverse, and yield-related traits still vary, but we can see a positive trend toward an increase. This diversity might be due to genetic background variations, which need further analysis. The traits need to be further validated under field conditions to elucidate the cause of the spikelet increase.

## Data Availability

The original contributions presented in the study are included in the article/Supplementary Material; further inquiries can be directed to the corresponding author.
