# Supplementary figures and images for: CRISPR-mediated promoter editing of a cis-regulatory element of OsNAS2 increases Zn uptake/translocation and plant yield in rice
Source: Front Genome Ed. 2024 Jan 23;5:1308228. doi: 10.3389/fgeed.2023.1308228 (PMC10844396; doi:10.3389/fgeed.2023.1308228)

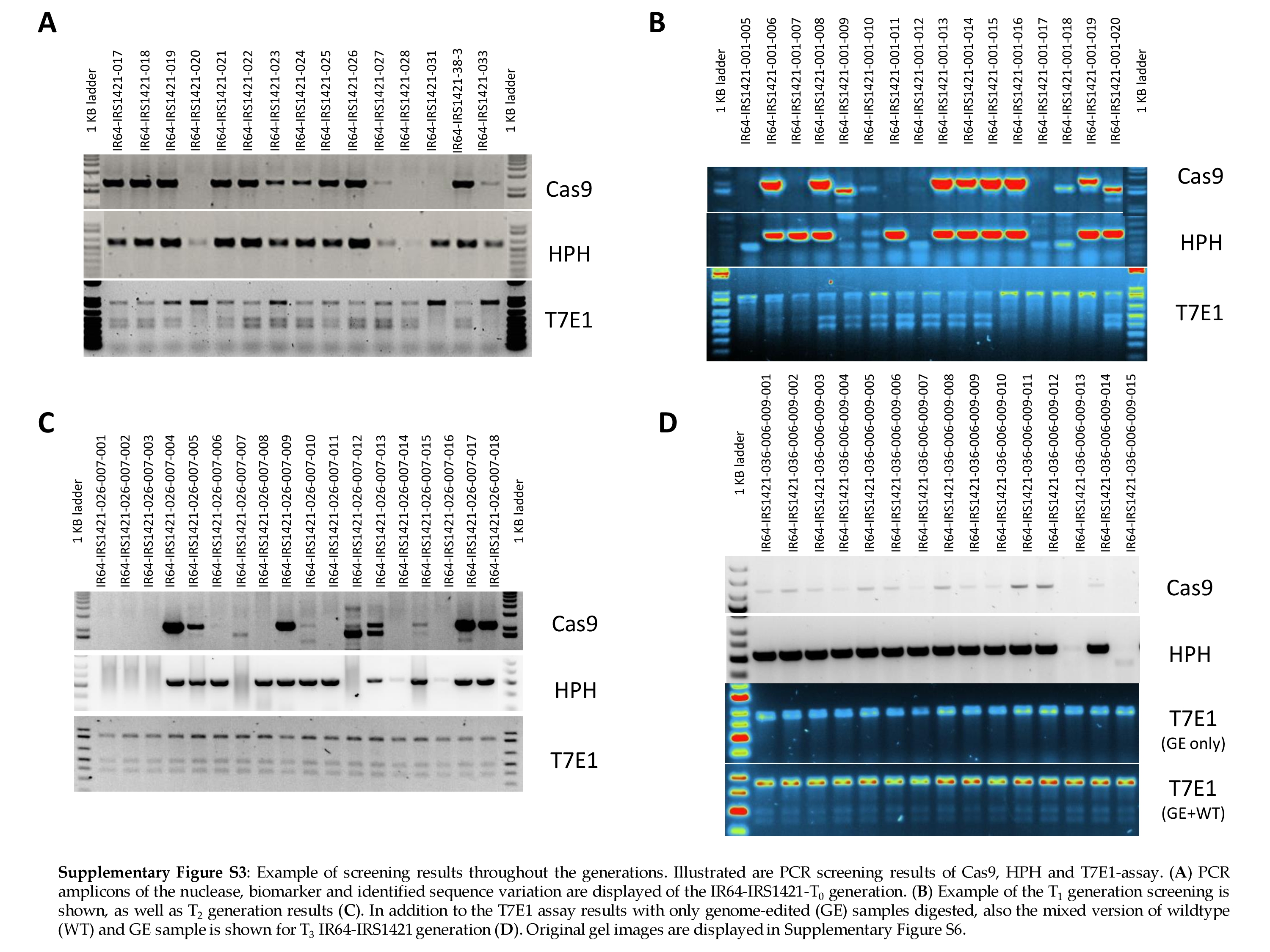

Supplement: Supplementary file 1 [file Image3.JPEG]

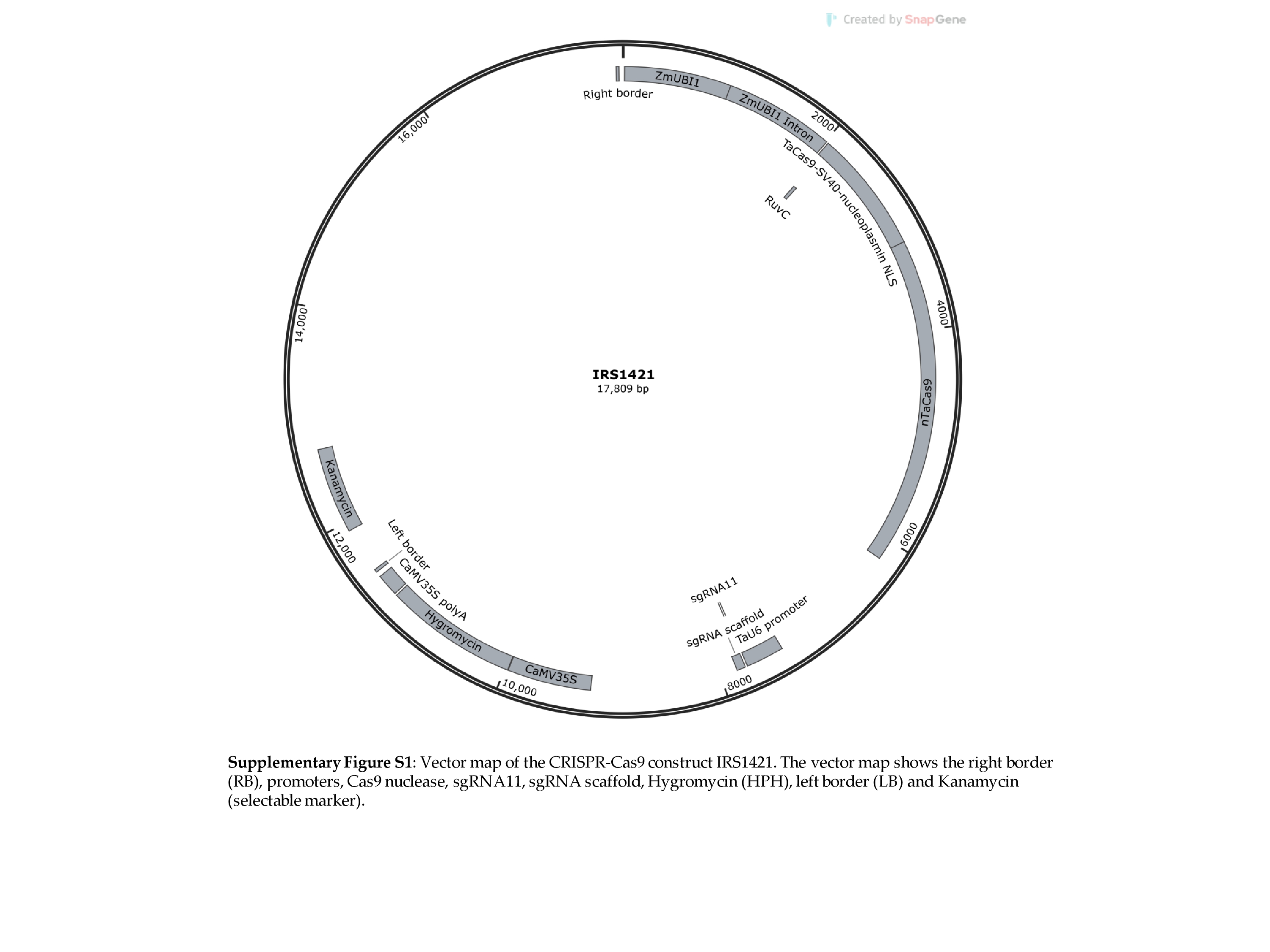

Supplement: Supplementary file 2 [file Image1.JPEG]

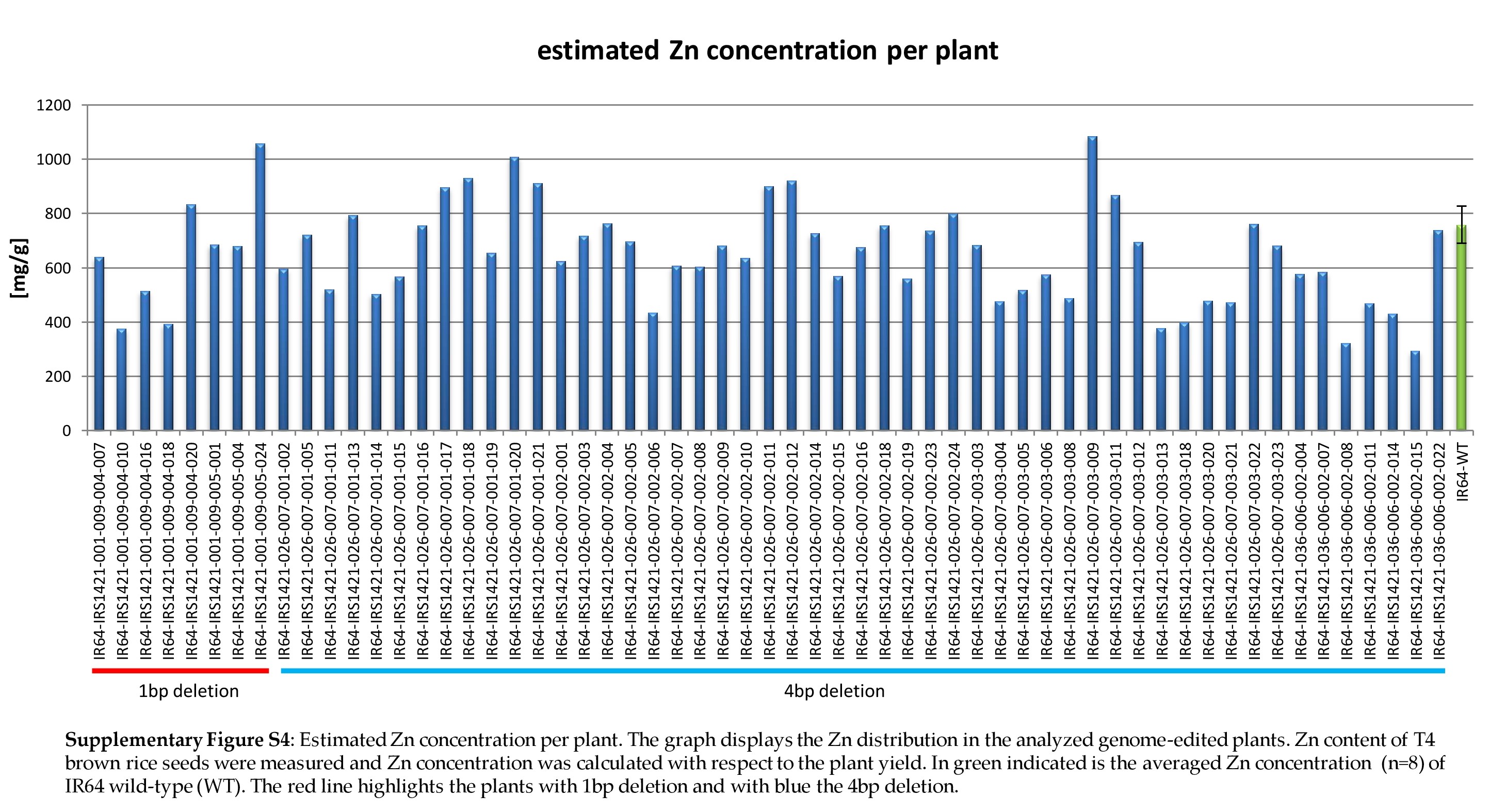

Supplement: Supplementary file 3 [file Image4.JPEG]

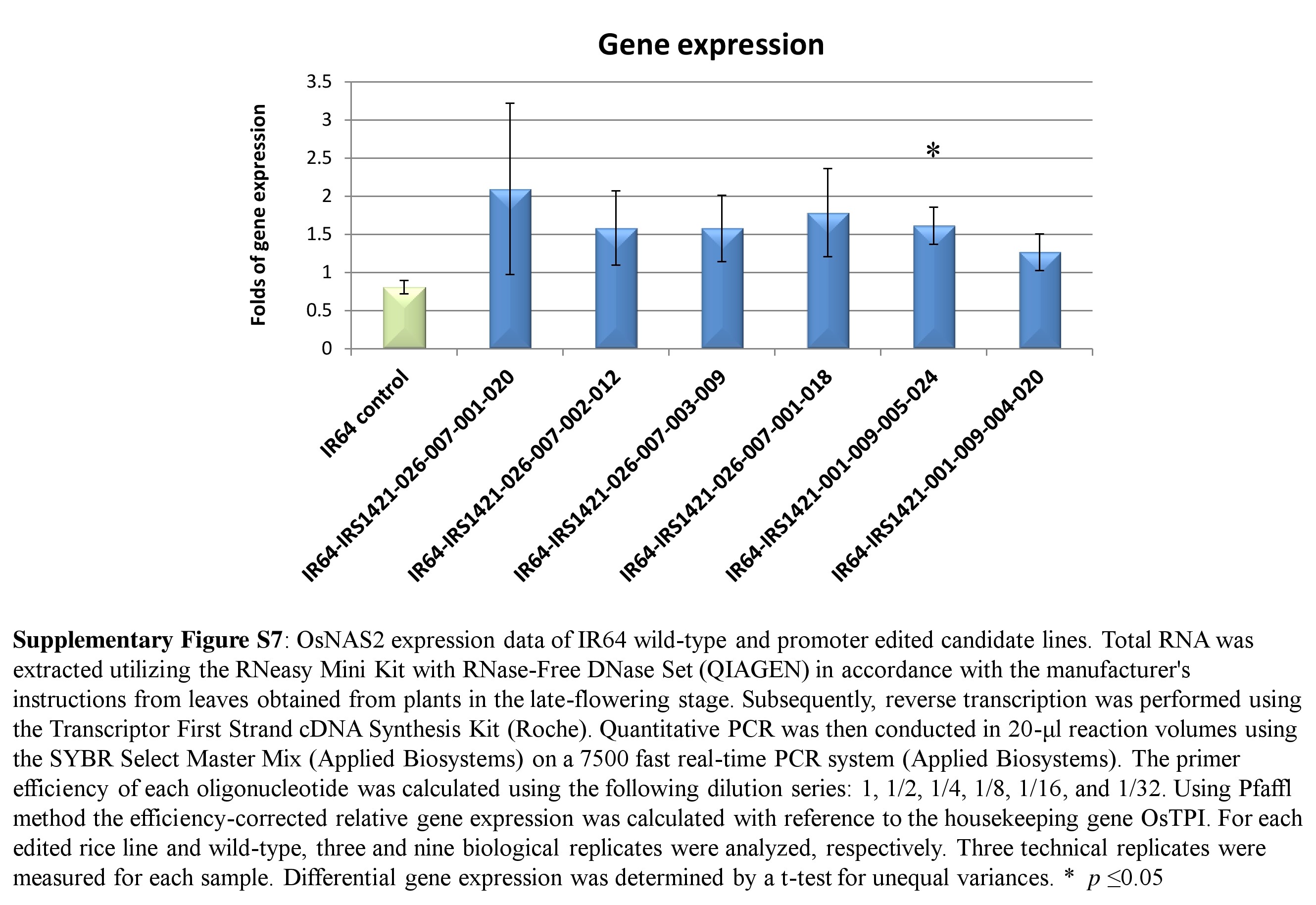

Supplement: Supplementary file 4 [file Image7.JPEG]

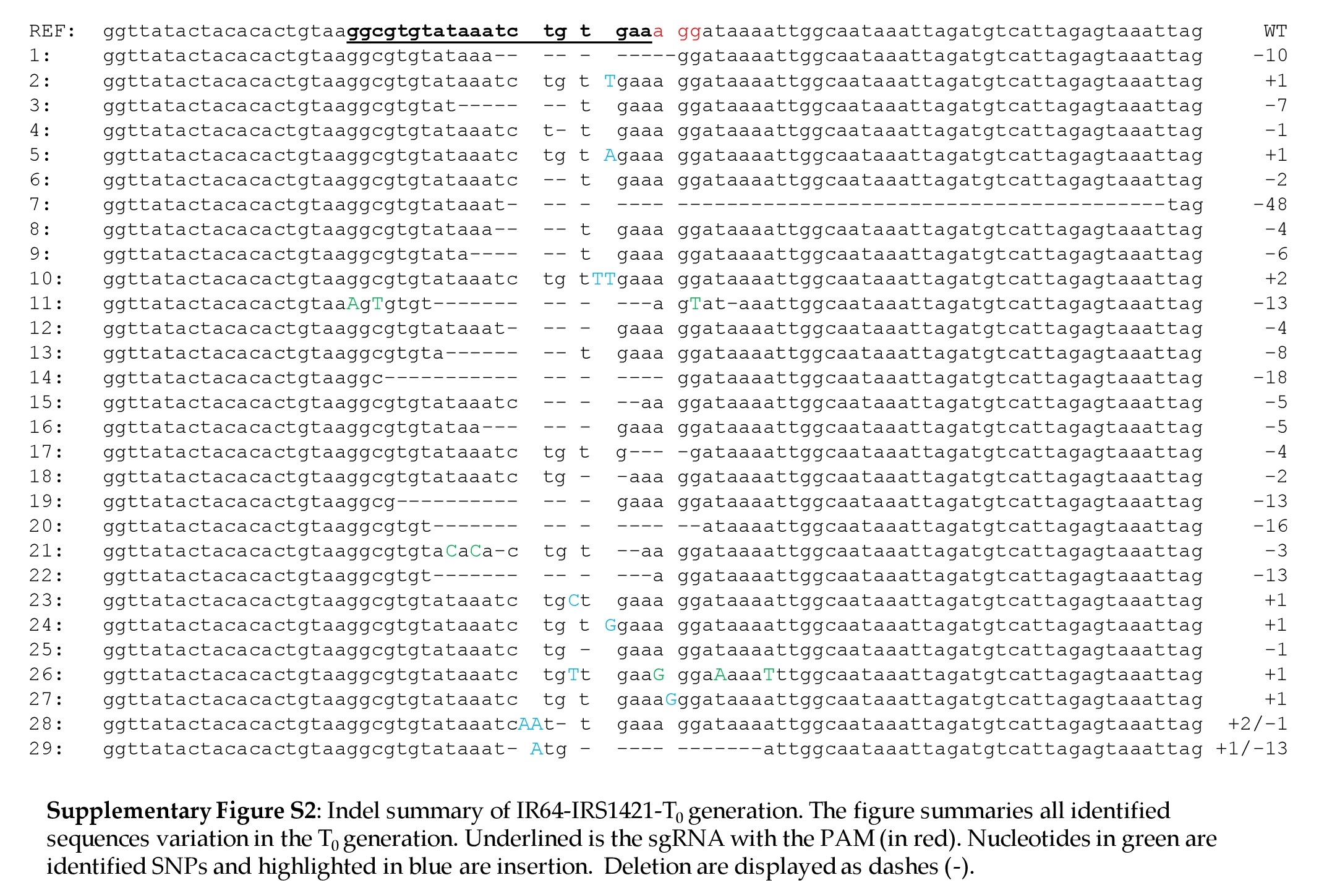

Supplement: Supplementary file 5 [file Image2.JPEG]

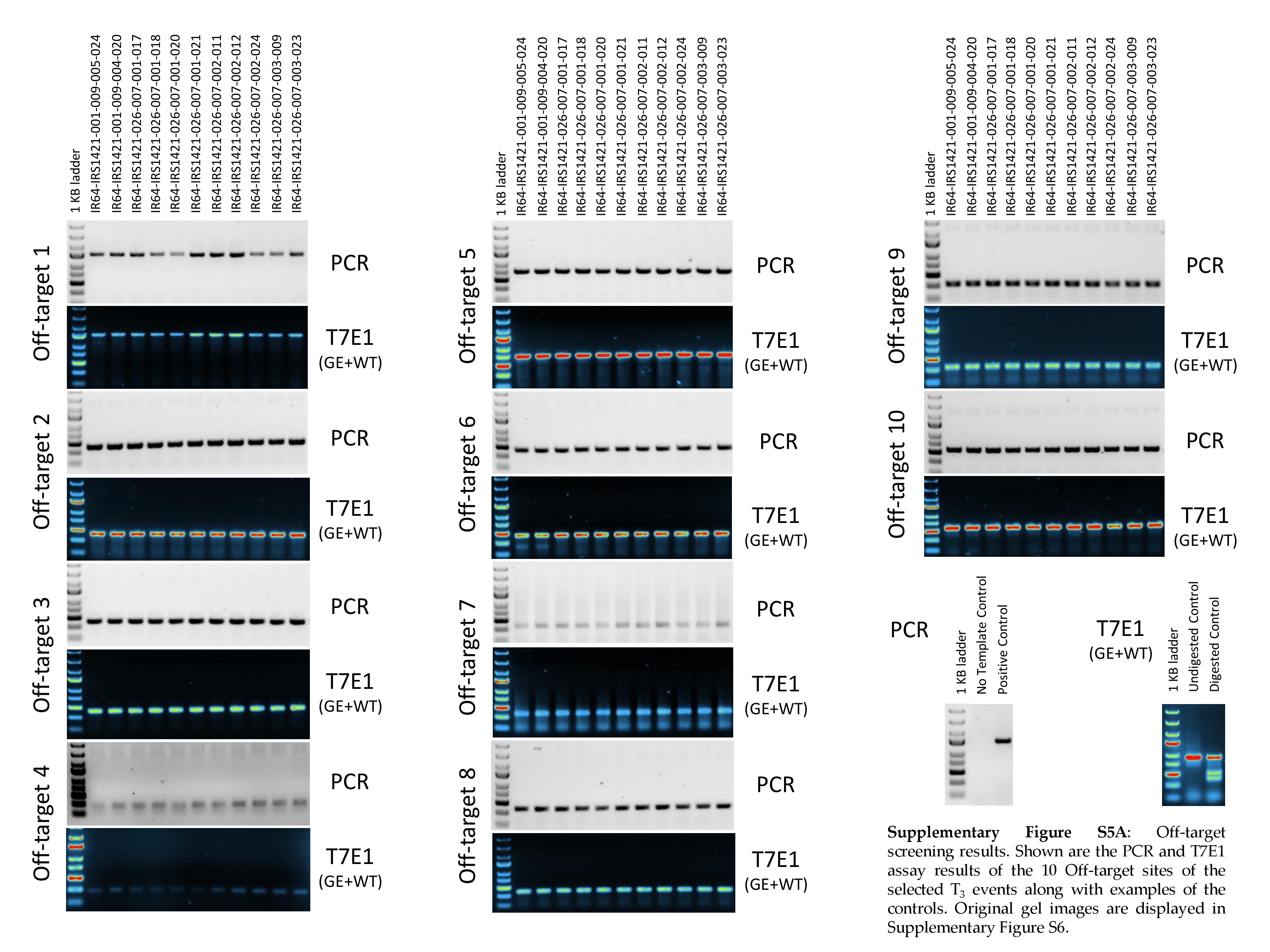

Supplement: Supplementary file 6 [file Image5.JPEG]

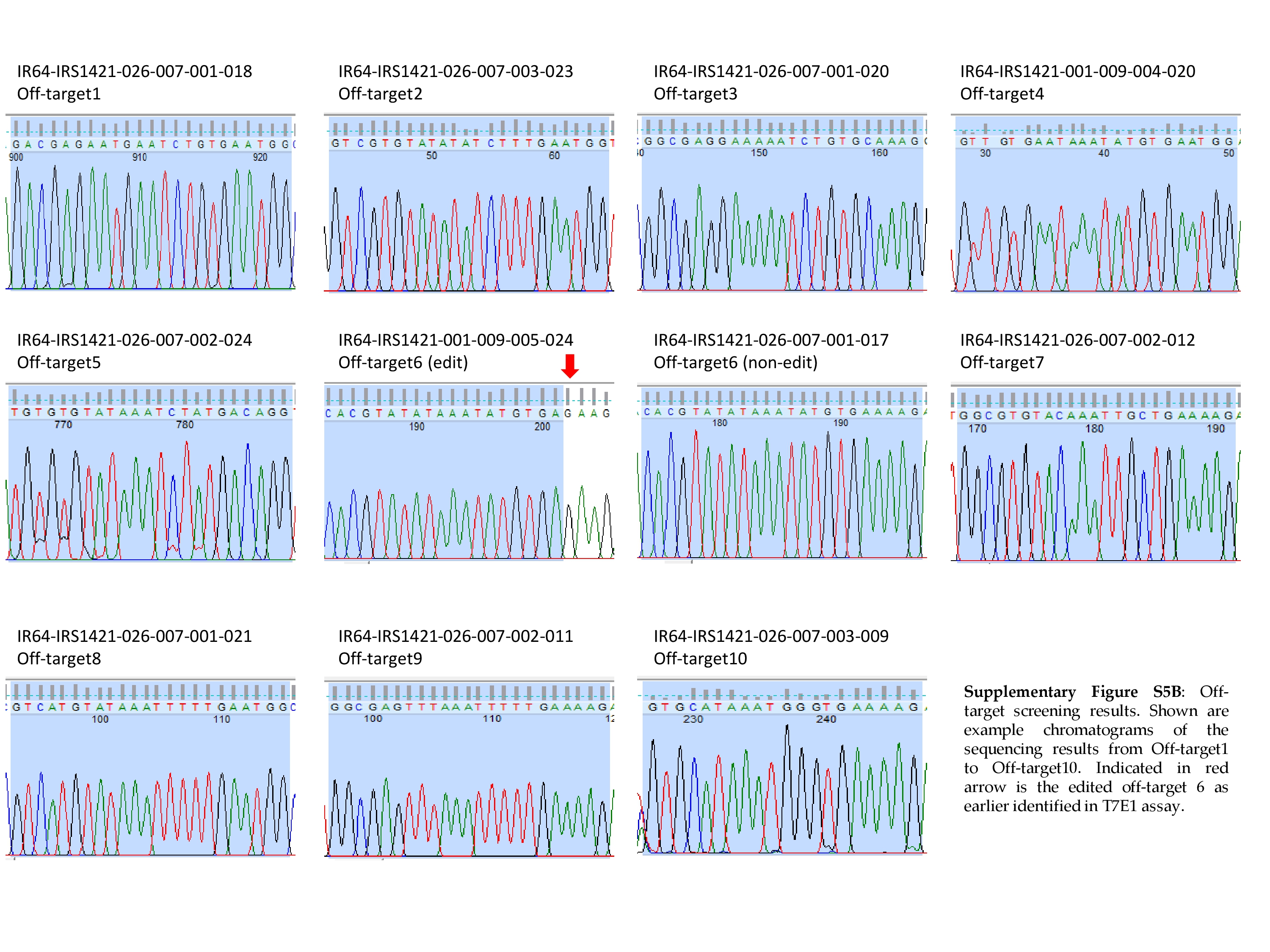

Supplement: Supplementary file 9 [file Image6.JPEG]
