# Supplementary material for: CRISPR-mediated promoter editing of a cis-regulatory element of OsNAS2 increases Zn uptake/translocation and plant yield in rice
Source: Front Genome Ed. 2024 Jan 23;5:1308228. doi: 10.3389/fgeed.2023.1308228 (PMC10844396; doi:10.3389/fgeed.2023.1308228)

Supplementary Figure S6: Compilation of original gel images, where cropped version were used in Supplementary Figure S2 and S4. Supplementary Figure S2 has the cropped version of gel image 1 to 11, displaying the Cas9, HPH screening as well as the T7E1 assay results from T_0 to T_3 generation. The gel images 12 to 22 are showing the PCR amplification of ROI for the specific Off-targets 1 to 10 (A) and the respective T7E1 assay result (B) of the T_3 generation. Indicated in frame are the cropped images displayed in Supplementary Figures S3 or S5 as well as the Plant-ID are displayed.

(1) HPH-screening IR64-IRS1421-T_0 generation

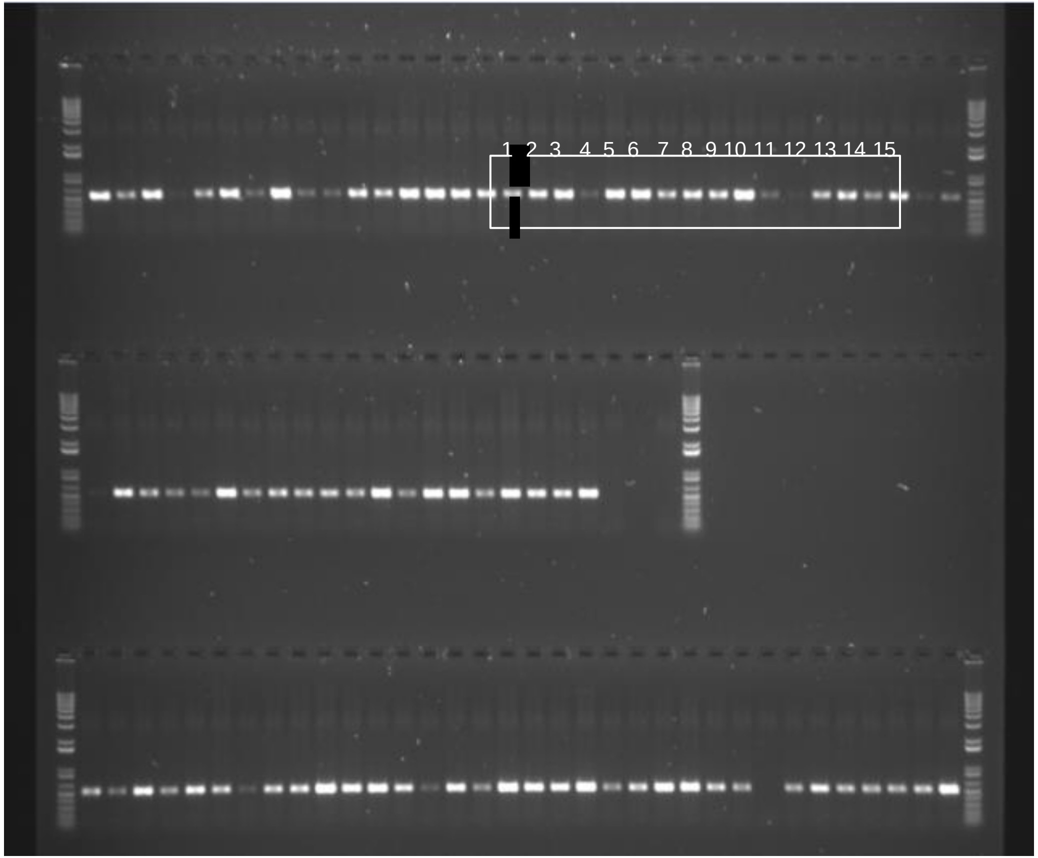

Sample	ID
1	IR64-IRS1421-017
2	IR64-IRS1421-018
3	IR64-IRS1421-019
4	IR64-IRS1421-020
5	IR64-IRS1421-021
6	IR64-IRS1421-022
7	IR64-IRS1421-023
8	IR64-IRS1421-024
9	IR64-IRS1421-025
10	IR64-IRS1421-026
11	IR64-IRS1421-027
12	IR64-IRS1421-028
13	IR64-IRS1421-031
14	IR64-IRS1421-38-3
15	IR64-IRS1421-033

(2) Cas9-screening IR64-IRS1421-T_0 generation

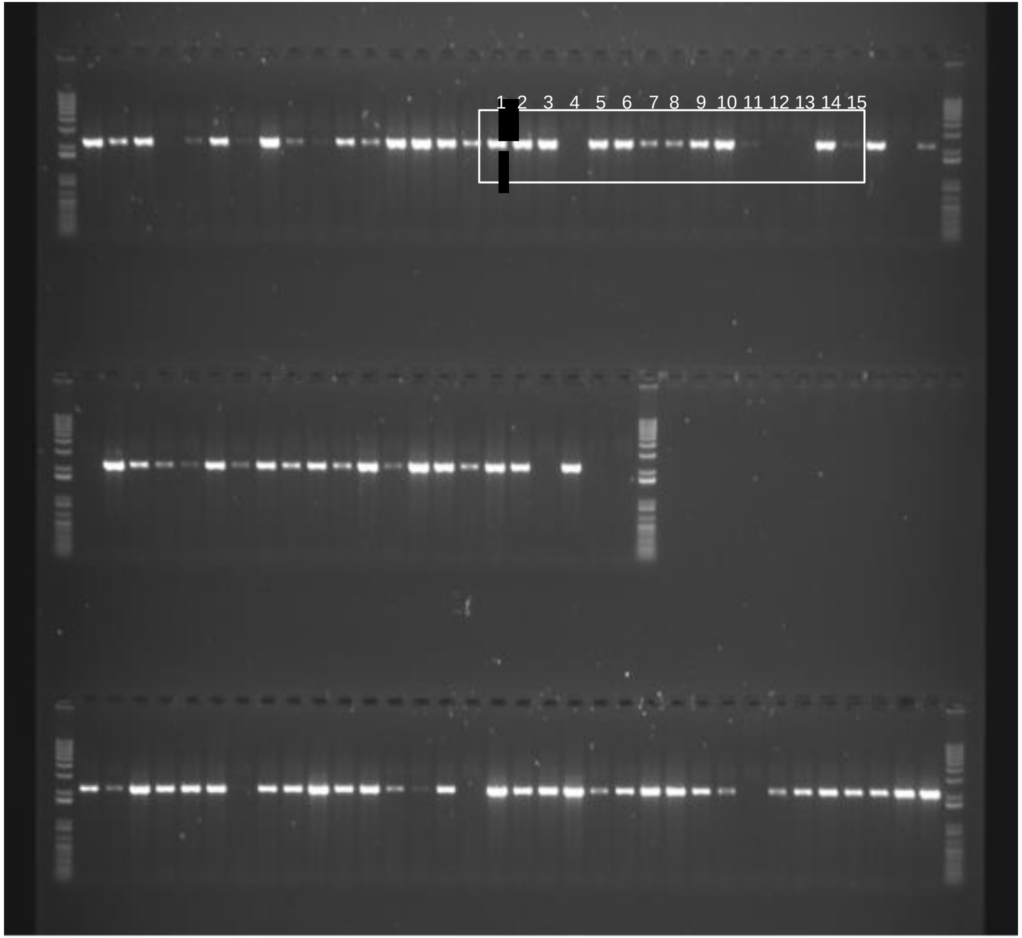

Sample	ID
1	IR64-IRS1421-017
2	IR64-IRS1421-018
3	IR64-IRS1421-019
4	IR64-IRS1421-020
5	IR64-IRS1421-021
6	IR64-IRS1421-022
7	IR64-IRS1421-023
8	IR64-IRS1421-024
9	IR64-IRS1421-025
10	IR64-IRS1421-026
11	IR64-IRS1421-027
12	IR64-IRS1421-028
13	IR64-IRS1421-031
14	IR64-IRS1421-38-3
15	IR64-IRS1421-033

(3) T7E1-screening IR64-IRS1421-T_0 generation

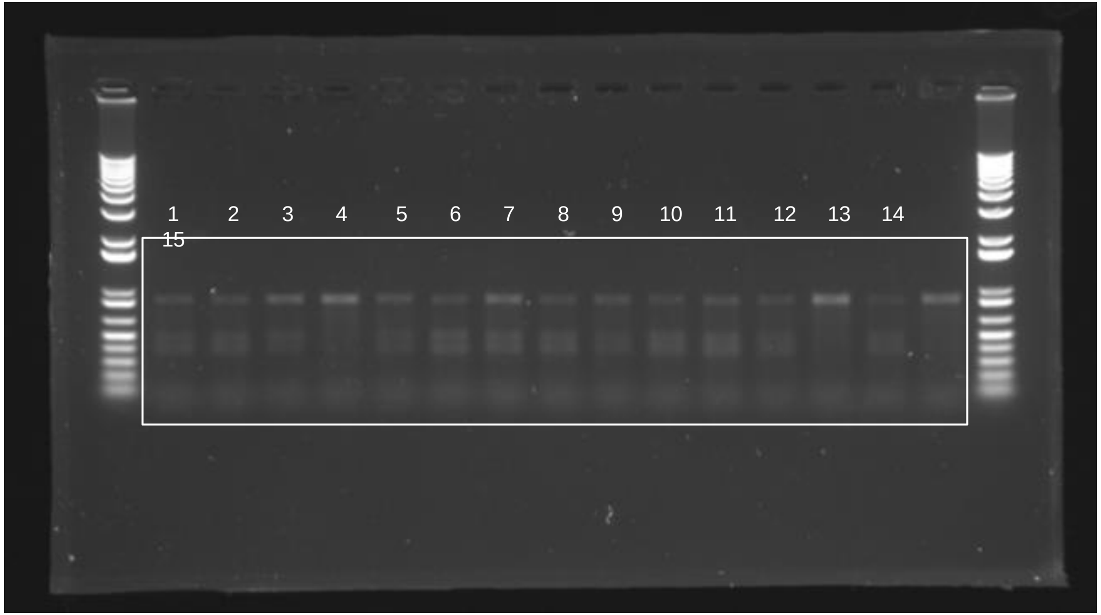

Sample	ID
1	IR64-IRS1421-017
2	IR64-IRS1421-018
3	IR64-IRS1421-019
4	IR64-IRS1421-020
5	IR64-IRS1421-021
6	IR64-IRS1421-022
7	IR64-IRS1421-023
8	IR64-IRS1421-024
9	IR64-IRS1421-025
10	IR64-IRS1421-026
11	IR64-IRS1421-027
12	IR64-IRS1421-028
13	IR64-IRS1421-031
14	IR64-IRS1421-38-3
15	IR64-IRS1421-033

(4) Cas9 (A) and HPH (B)-screening IR64-IRS1421-T_1 generation

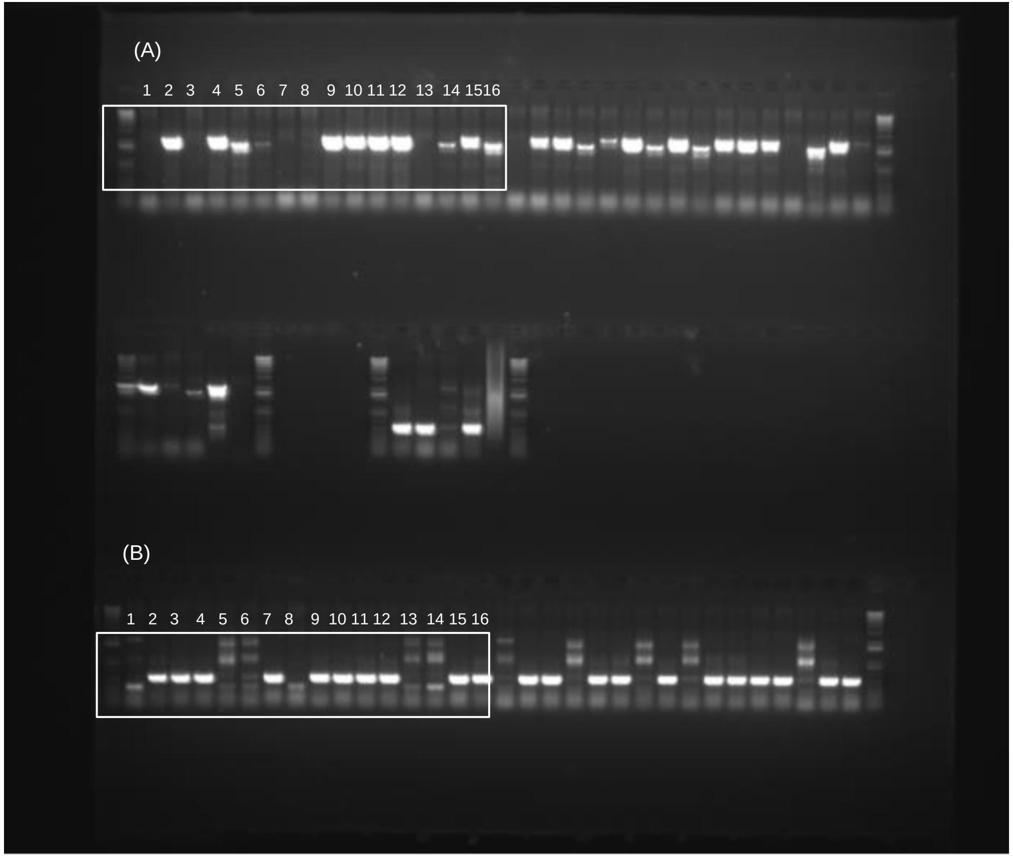

Sample	ID
1	IR64-IRS1421-001-005
2	IR64-IRS1421-001-006
3	IR64-IRS1421-001-007
4	IR64-IRS1421-001-008
5	IR64-IRS1421-001-009
6	IR64-IRS1421-001-010
7	IR64-IRS1421-001-011
8	IR64-IRS1421-001-012
9	IR64-IRS1421-001-013
10	IR64-IRS1421-001-014
11	IR64-IRS1421-001-015
12	IR64-IRS1421-001-016
13	IR64-IRS1421-001-017
14	IR64-IRS1421-001-018
15	IR64-IRS1421-001-019
16	IR64-IRS1421-001-020

(5) T7E1-screening IR64-IRS1421-T_1 generation

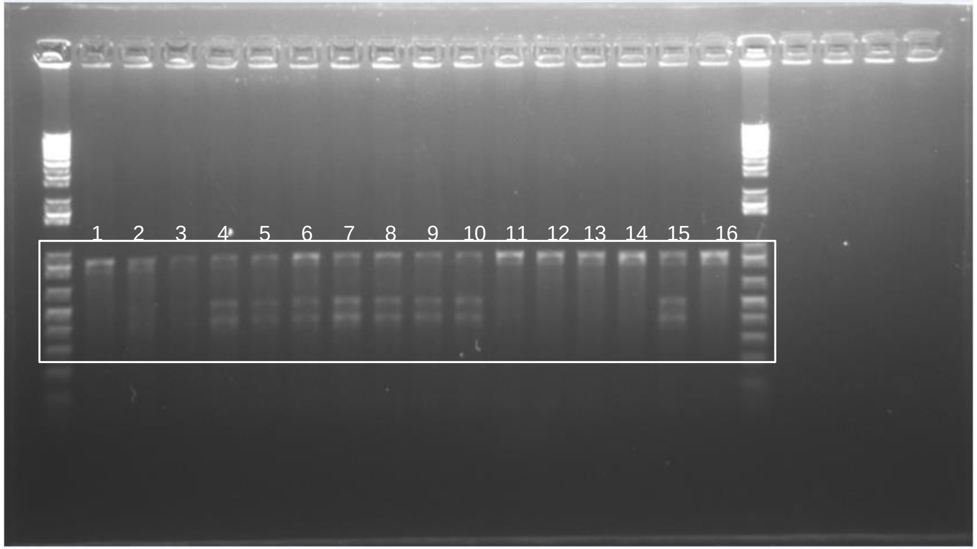

Sample	ID
1	IR64-IRS1421-001-005
2	IR64-IRS1421-001-006
3	IR64-IRS1421-001-007
4	IR64-IRS1421-001-008
5	IR64-IRS1421-001-009
6	IR64-IRS1421-001-010
7	IR64-IRS1421-001-011
8	IR64-IRS1421-001-012
9	IR64-IRS1421-001-013
10	IR64-IRS1421-001-014
11	IR64-IRS1421-001-015
12	IR64-IRS1421-001-016
13	IR64-IRS1421-001-017
14	IR64-IRS1421-001-018
15	IR64-IRS1421-001-019
16	IR64-IRS1421-001-020

(6) HPH-screening IR64-IRS1421-T_2 generation

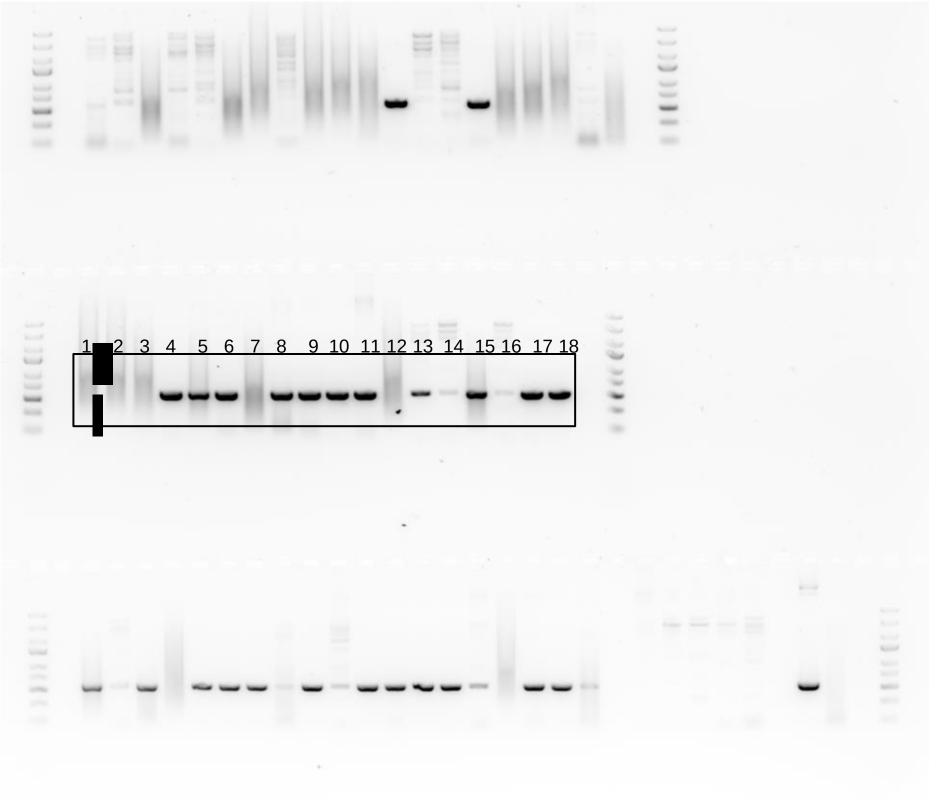

Sample	ID
1	IR64-IRS1421-026-007-001
2	IR64-IRS1421-026-007-002
3	IR64-IRS1421-026-007-003
4	IR64-IRS1421-026-007-004
5	IR64-IRS1421-026-007-005
6	IR64-IRS1421-026-007-006
7	IR64-IRS1421-026-007-007
8	IR64-IRS1421-026-007-008
9	IR64-IRS1421-026-007-009
10	IR64-IRS1421-026-007-010
11	IR64-IRS1421-026-007-011
12	IR64-IRS1421-026-007-012
13	IR64-IRS1421-026-007-013
14	IR64-IRS1421-026-007-014
15	IR64-IRS1421-026-007-015
16	IR64-IRS1421-026-007-016
17	IR64-IRS1421-026-007-017
18	IR64-IRS1421-026-007-018

(7)Cas9-screening IR64-IRS1421-T_2 generation

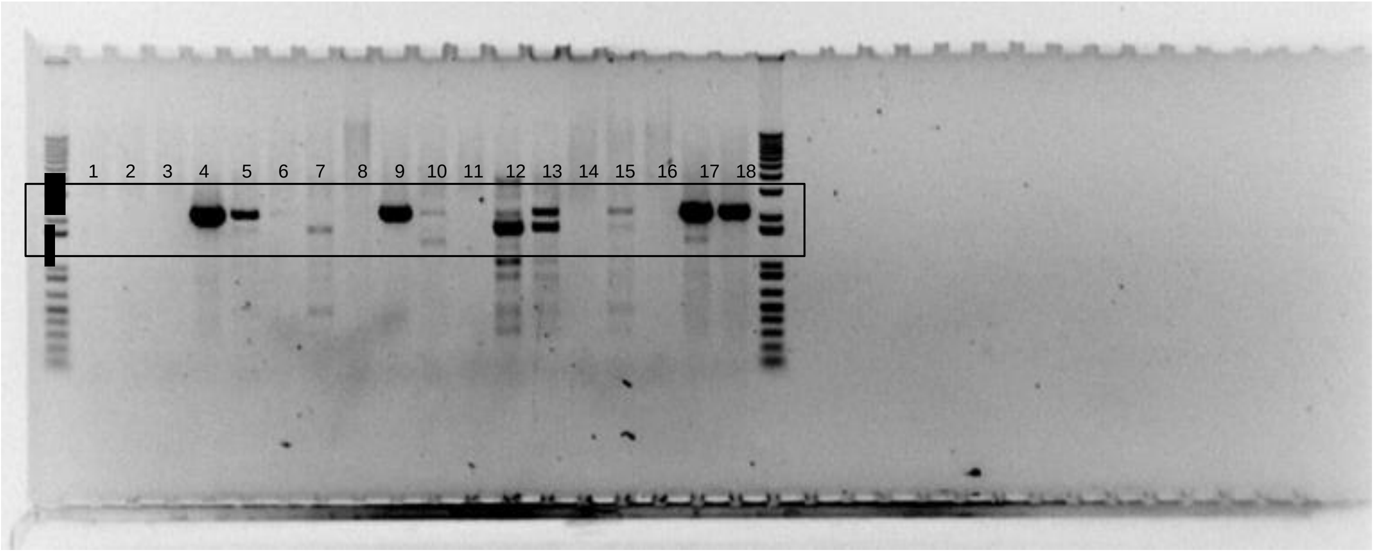

Sample	ID
1	IR64-IRS1421-026-007-001
2	IR64-IRS1421-026-007-002
3	IR64-IRS1421-026-007-003
4	IR64-IRS1421-026-007-004
5	IR64-IRS1421-026-007-005
6	IR64-IRS1421-026-007-006
7	IR64-IRS1421-026-007-007
8	IR64-IRS1421-026-007-008
9	IR64-IRS1421-026-007-009
10	IR64-IRS1421-026-007-010
11	IR64-IRS1421-026-007-011
12	IR64-IRS1421-026-007-012
13	IR64-IRS1421-026-007-013
14	IR64-IRS1421-026-007-014
15	IR64-IRS1421-026-007-015
16	IR64-IRS1421-026-007-016
17	IR64-IRS1421-026-007-017
18	IR64-IRS1421-026-007-018

(8)T7E1-screening IR64-IRS1421-T_2 generation

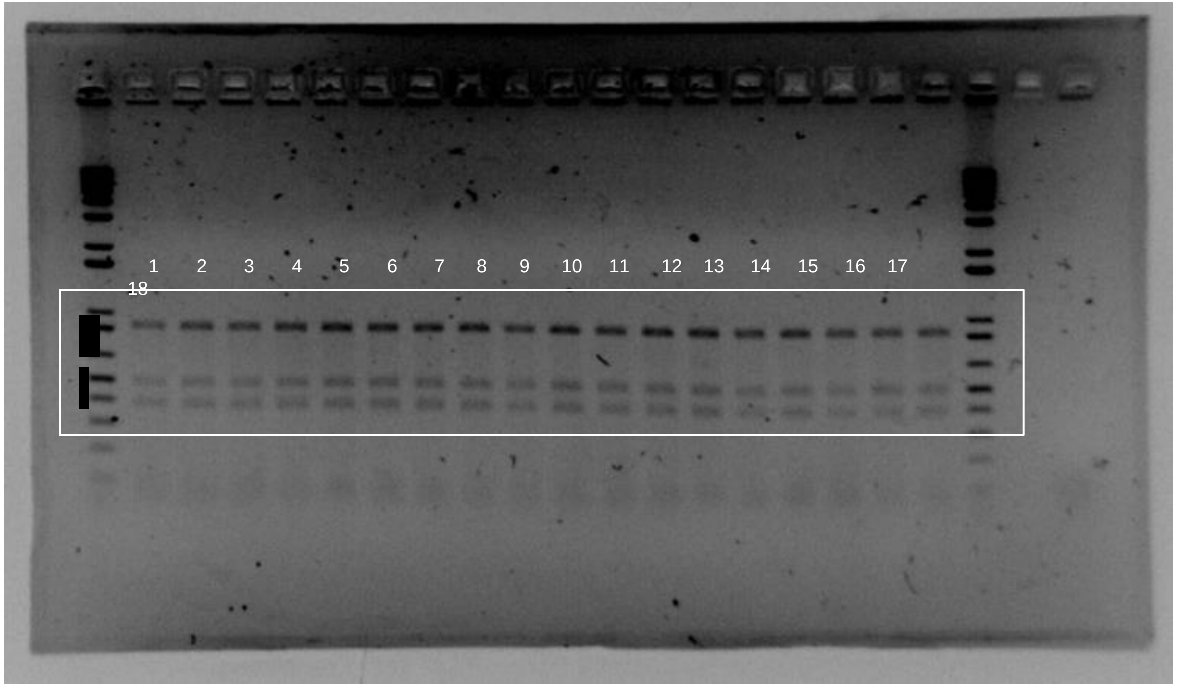

Sample	ID
1	IR64-IRS1421-026-007-001
2	IR64-IRS1421-026-007-002
3	IR64-IRS1421-026-007-003
4	IR64-IRS1421-026-007-004
5	IR64-IRS1421-026-007-005
6	IR64-IRS1421-026-007-006
7	IR64-IRS1421-026-007-007
8	IR64-IRS1421-026-007-008
9	IR64-IRS1421-026-007-009
10	IR64-IRS1421-026-007-010
11	IR64-IRS1421-026-007-011
12	IR64-IRS1421-026-007-012
13	IR64-IRS1421-026-007-013
14	IR64-IRS1421-026-007-014
15	IR64-IRS1421-026-007-015
16	IR64-IRS1421-026-007-016
17	IR64-IRS1421-026-007-017
18	IR64-IRS1421-026-007-018

(9) HPH-screening IR64-IRS1421-T_3 generation

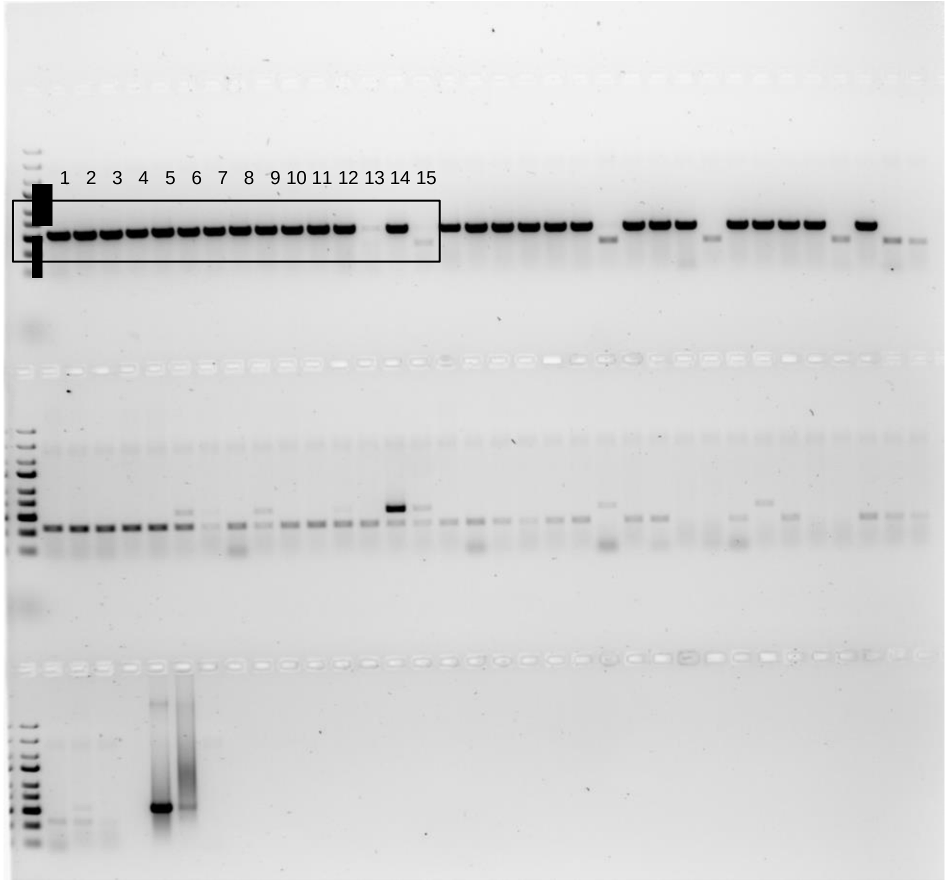

Sample	ID
1	IR64-IRS1421-036-006-009-001
2	IR64-IRS1421-036-006-009-002
3	IR64-IRS1421-036-006-009-003
4	IR64-IRS1421-036-006-009-004
5	IR64-IRS1421-036-006-009-005
6	IR64-IRS1421-036-006-009-006
7	IR64-IRS1421-036-006-009-007
8	IR64-IRS1421-036-006-009-008
9	IR64-IRS1421-036-006-009-009
10	IR64-IRS1421-036-006-009-010
11	IR64-IRS1421-036-006-009-011
12	IR64-IRS1421-036-006-009-012
13	IR64-IRS1421-036-006-009-013
14	IR64-IRS1421-036-006-009-014
15	IR64-IRS1421-036-006-009-015

(10) Cas9-screening IR64-IRS1421-T_3 generation

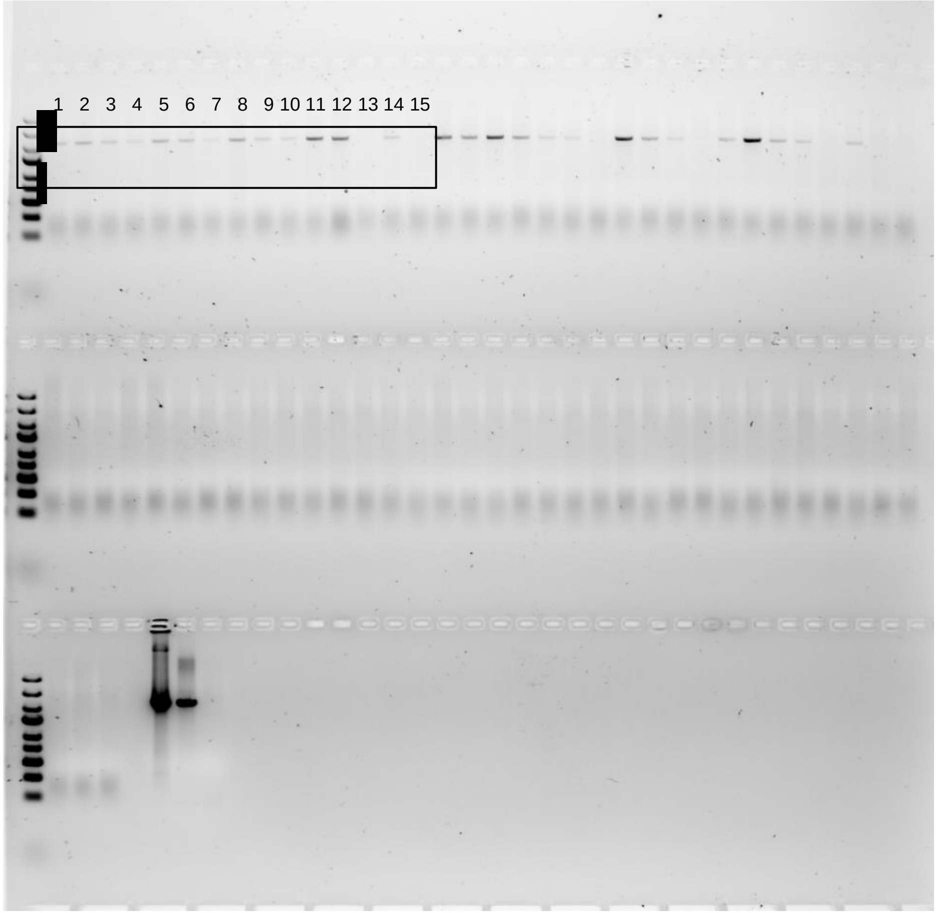

Sample	ID
1	IR64-IRS1421-036-006-009-001
2	IR64-IRS1421-036-006-009-002
3	IR64-IRS1421-036-006-009-003
4	IR64-IRS1421-036-006-009-004
5	IR64-IRS1421-036-006-009-005
6	IR64-IRS1421-036-006-009-006
7	IR64-IRS1421-036-006-009-007
8	IR64-IRS1421-036-006-009-008
9	IR64-IRS1421-036-006-009-009
10	IR64-IRS1421-036-006-009-010
11	IR64-IRS1421-036-006-009-011
12	IR64-IRS1421-036-006-009-012
13	IR64-IRS1421-036-006-009-013
14	IR64-IRS1421-036-006-009-014
15	IR64-IRS1421-036-006-009-015

(11) T7E1-screening IR64-IRS1421-T_3 generation (GE only)

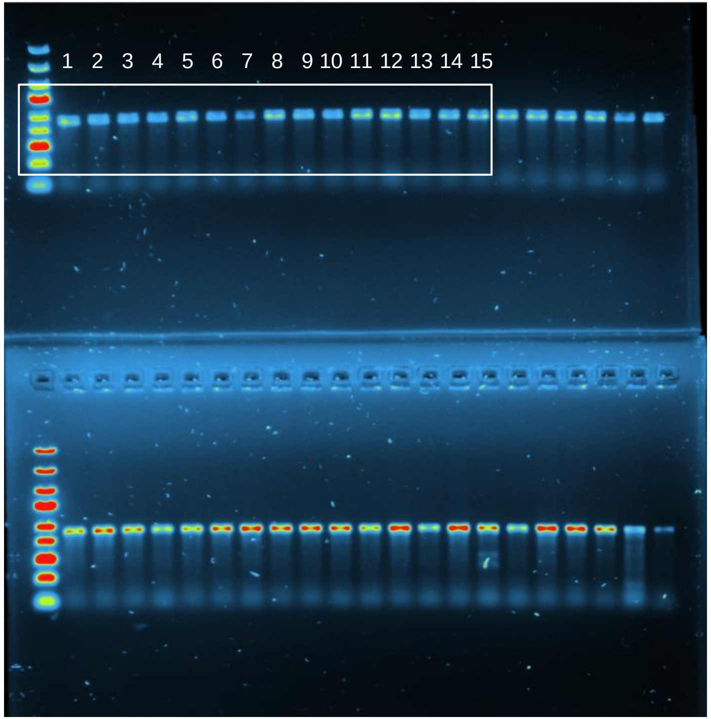

Sample	ID
1	IR64-IRS1421-036-006-009-001
2	IR64-IRS1421-036-006-009-002
3	IR64-IRS1421-036-006-009-003
4	IR64-IRS1421-036-006-009-004
5	IR64-IRS1421-036-006-009-005
6	IR64-IRS1421-036-006-009-006
7	IR64-IRS1421-036-006-009-007
8	IR64-IRS1421-036-006-009-008
9	IR64-IRS1421-036-006-009-009
10	IR64-IRS1421-036-006-009-010
11	IR64-IRS1421-036-006-009-011
12	IR64-IRS1421-036-006-009-012
13	IR64-IRS1421-036-006-009-013
14	IR64-IRS1421-036-006-009-014
15	IR64-IRS1421-036-006-009-015

(12) T7E1-screening IR64-IRS1421-T_3 generation (GE+Wt)

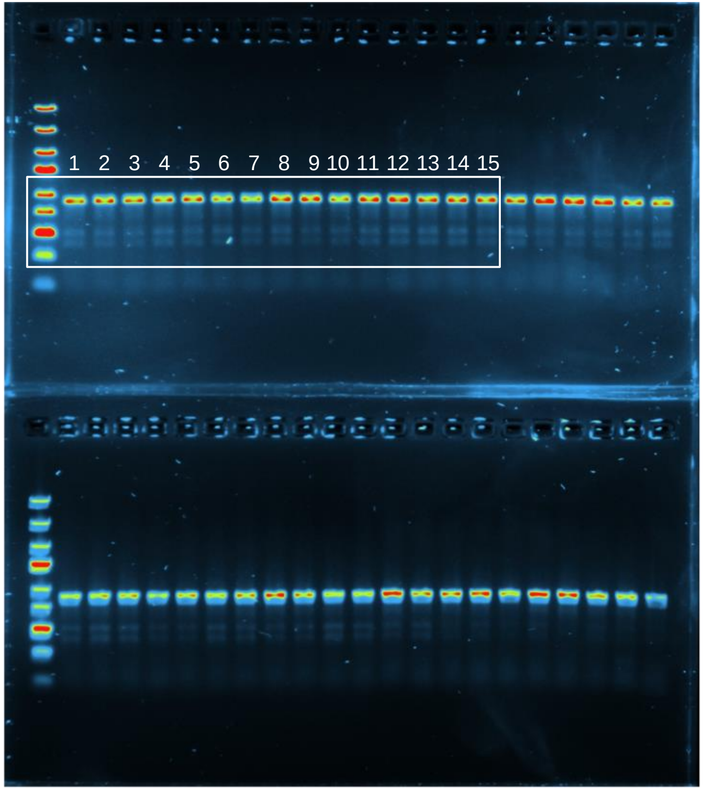

Sample	ID
1	IR64-IRS1421-036-006-009-001
2	IR64-IRS1421-036-006-009-002
3	IR64-IRS1421-036-006-009-003
4	IR64-IRS1421-036-006-009-004
5	IR64-IRS1421-036-006-009-005
6	IR64-IRS1421-036-006-009-006
7	IR64-IRS1421-036-006-009-007
8	IR64-IRS1421-036-006-009-008
9	IR64-IRS1421-036-006-009-009
10	IR64-IRS1421-036-006-009-010
11	IR64-IRS1421-036-006-009-011
12	IR64-IRS1421-036-006-009-012
13	IR64-IRS1421-036-006-009-013
14	IR64-IRS1421-036-006-009-014
15	IR64-IRS1421-036-006-009-015

(13)Off-target Screening IR64-IRS1421-T_3 generation

Off-target 1 PCR (A) and T7E1 assay (B) results found in Supplementary 5

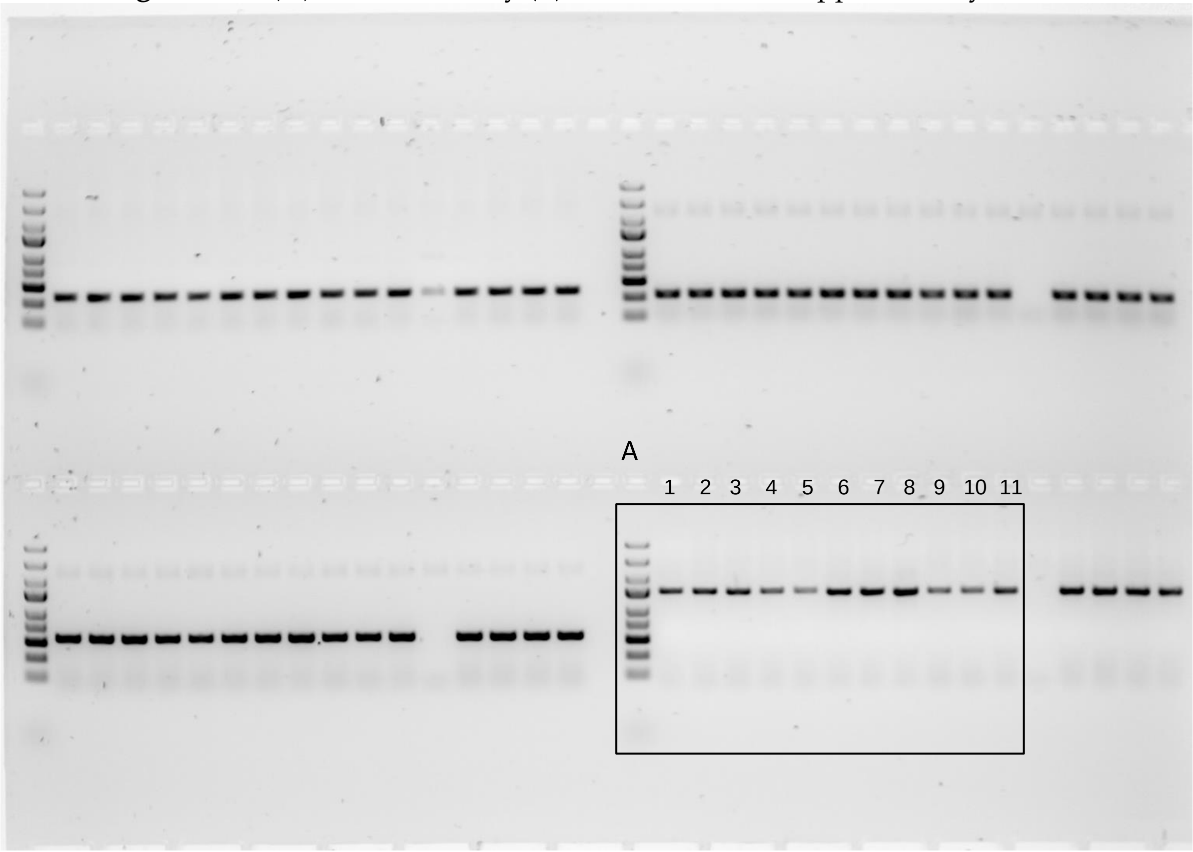

Sample	ID
1	IR64-IRS1421-001-009-005-024
2	IR64-IRS1421-001-009-004-020
3	IR64-IRS1421-026-007-001-017
4	IR64-IRS1421-026-007-001-018
5	IR64-IRS1421-026-007-001-020
6	IR64-IRS1421-026-007-001-021
7	IR64-IRS1421-026-007-002-011
8	IR64-IRS1421-026-007-002-012
9	IR64-IRS1421-026-007-002-024
10	IR64-IRS1421-026-007-003-009
11	IR64-IRS1421-026-007-003-023

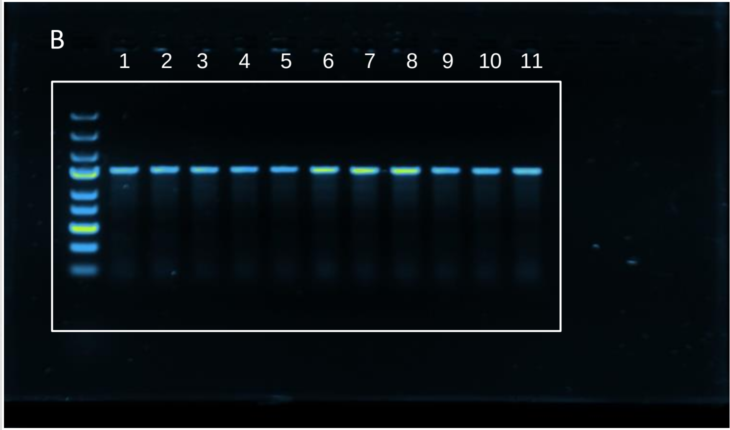

(14) Off-target Screening IR64-IRS1421-T_3 generation

Off-target 2 PCR (A) and T7E1 assay (B) results found in Supplementary 5

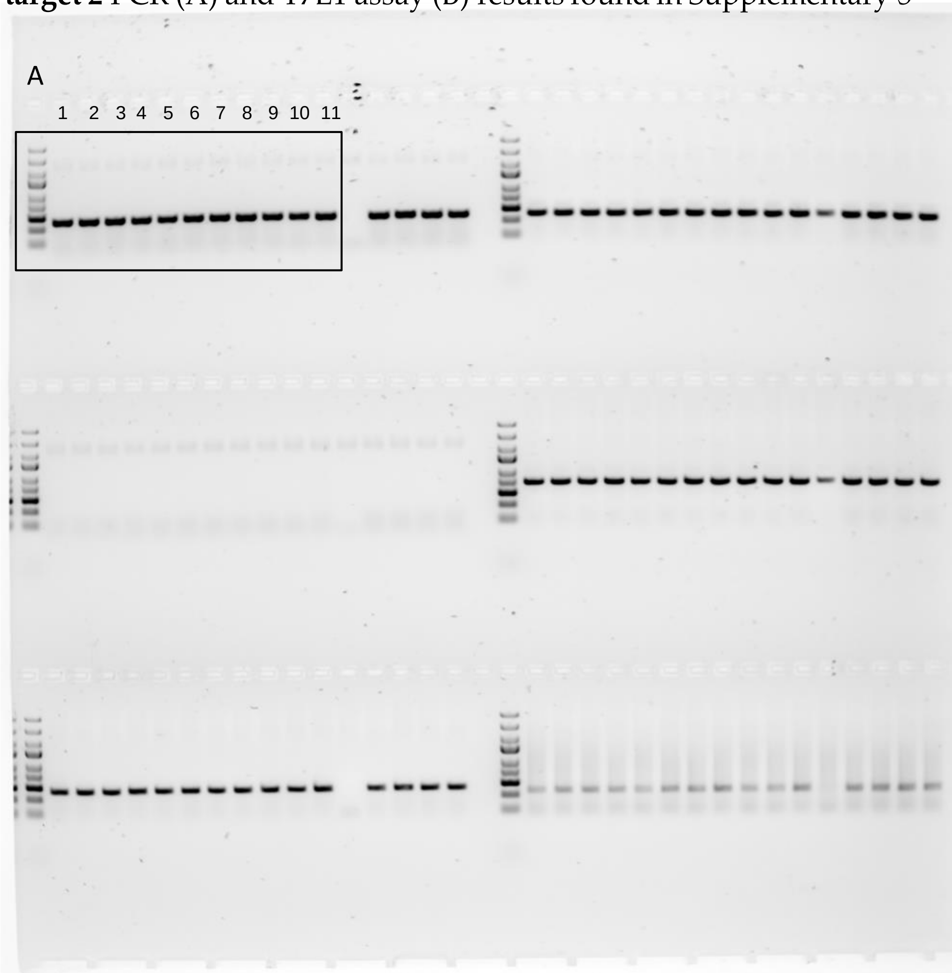

Sample	ID
1	IR64-IRS1421-001-009-005-024
2	IR64-IRS1421-001-009-004-020
3	IR64-IRS1421-026-007-001-017
4	IR64-IRS1421-026-007-001-018
5	IR64-IRS1421-026-007-001-020
6	IR64-IRS1421-026-007-001-021
7	IR64-IRS1421-026-007-002-011
8	IR64-IRS1421-026-007-002-012
9	IR64-IRS1421-026-007-002-024
10	IR64-IRS1421-026-007-003-009
11	IR64-IRS1421-026-007-003-023

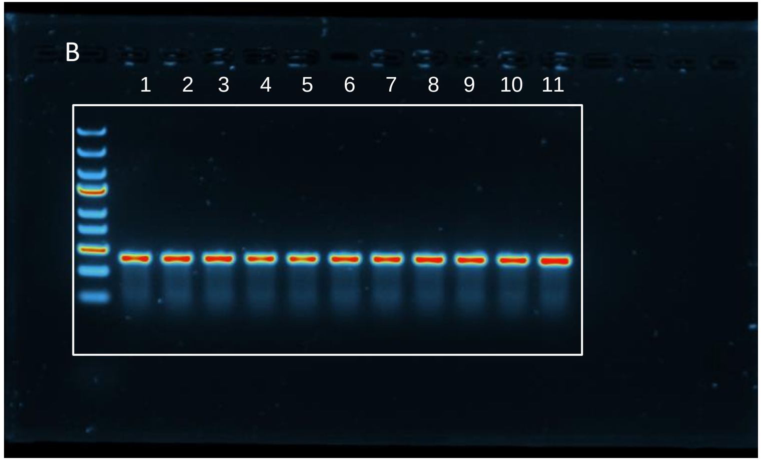

(15) Off-target Screening IR64-IRS1421-T_3 generation

Off-target 3 PCR (A) and T7E1 assay (B) results found in Supplementary 5

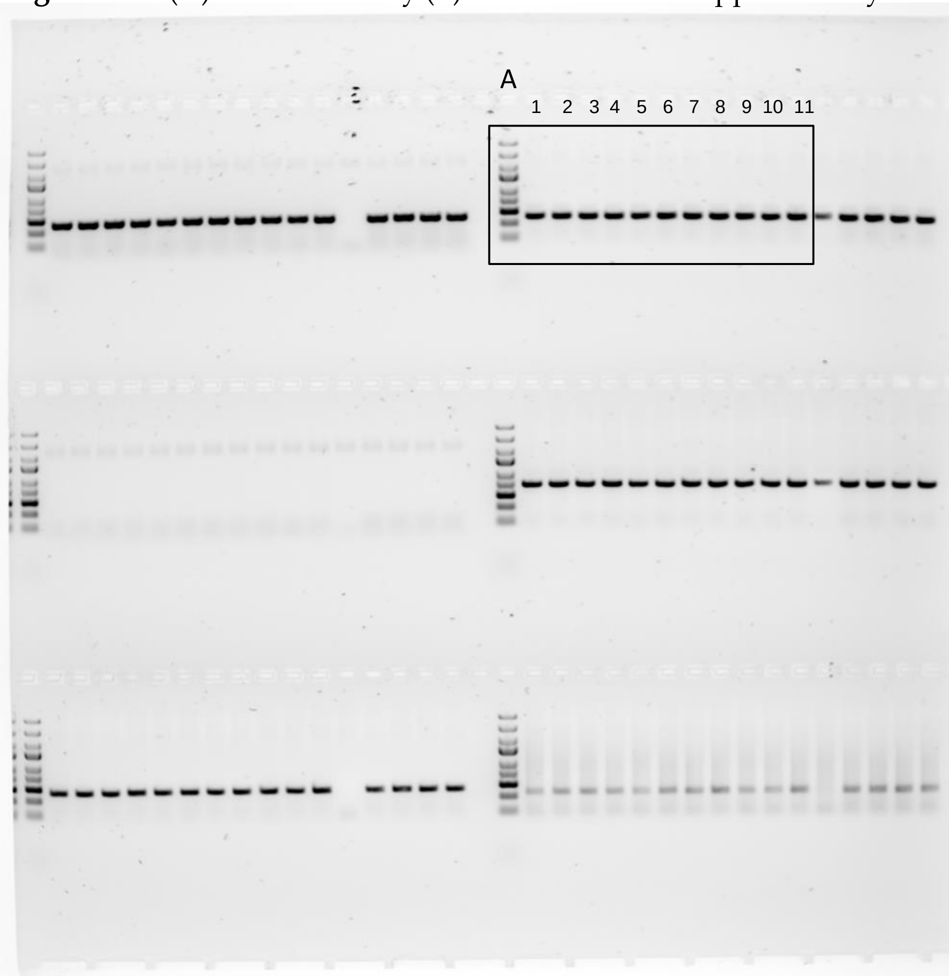

Sample	ID
1	IR64-IRS1421-001-009-005-024
2	IR64-IRS1421-001-009-004-020
3	IR64-IRS1421-026-007-001-017
4	IR64-IRS1421-026-007-001-018
5	IR64-IRS1421-026-007-001-020
6	IR64-IRS1421-026-007-001-021
7	IR64-IRS1421-026-007-002-011
8	IR64-IRS1421-026-007-002-012
9	IR64-IRS1421-026-007-002-024
10	IR64-IRS1421-026-007-003-009
11	IR64-IRS1421-026-007-003-023

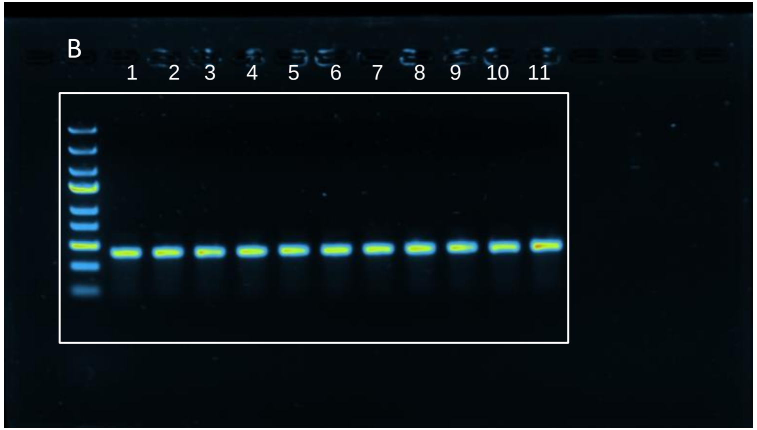

(16) Off-target Screening IR64-IRS1421-T_3 generation

Off-target 4 PCR (A) and T7E1 assay (B) results found in Supplementary 5

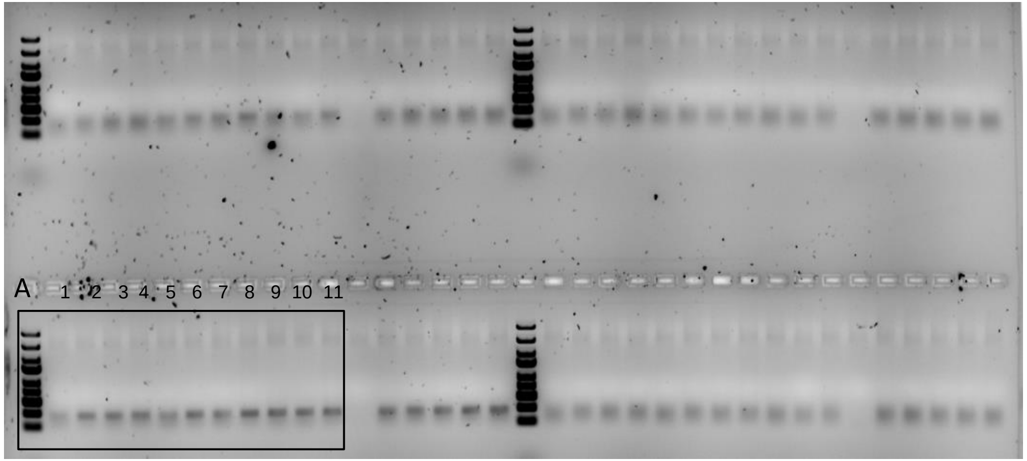

Sample	ID
1	IR64-IRS1421-001-009-005-024
2	IR64-IRS1421-001-009-004-020
3	IR64-IRS1421-026-007-001-017
4	IR64-IRS1421-026-007-001-018
5	IR64-IRS1421-026-007-001-020
6	IR64-IRS1421-026-007-001-021
7	IR64-IRS1421-026-007-002-011
8	IR64-IRS1421-026-007-002-012
9	IR64-IRS1421-026-007-002-024
10	IR64-IRS1421-026-007-003-009
11	IR64-IRS1421-026-007-003-023

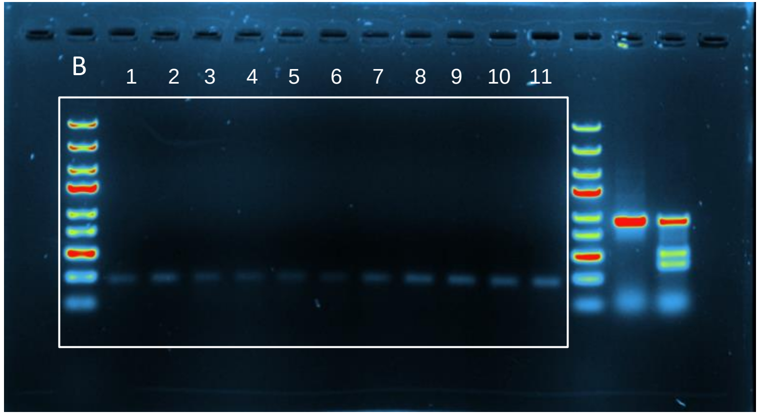

(17) Off-target Screening IR64-IRS1421-T_3 generation

Off-target 5 PCR (A) and T7E1 assay (B) results found in Supplementary 5

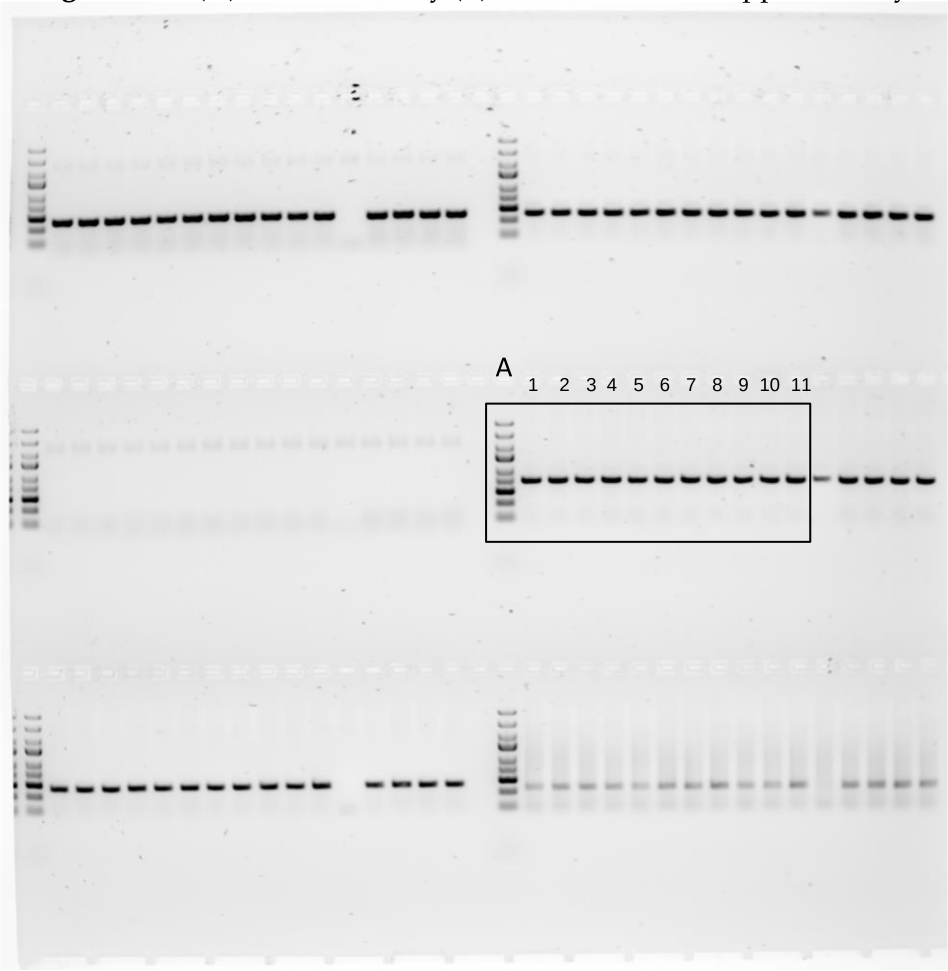

Sample	ID
1	IR64-IRS1421-001-009-005-024
2	IR64-IRS1421-001-009-004-020
3	IR64-IRS1421-026-007-001-017
4	IR64-IRS1421-026-007-001-018
5	IR64-IRS1421-026-007-001-020
6	IR64-IRS1421-026-007-001-021
7	IR64-IRS1421-026-007-002-011
8	IR64-IRS1421-026-007-002-012
9	IR64-IRS1421-026-007-002-024
10	IR64-IRS1421-026-007-003-009
11	IR64-IRS1421-026-007-003-023

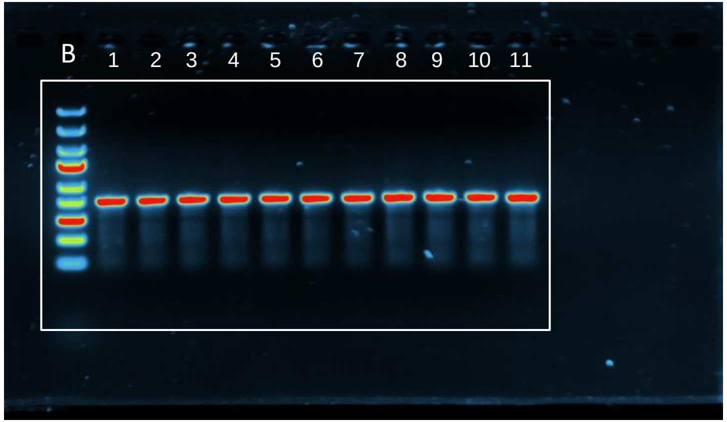

(18) Off-target Screening IR64-IRS1421-T_3 generation

Off-target 6 PCR (A) and T7E1 assay (B) results found in Supplementary 5

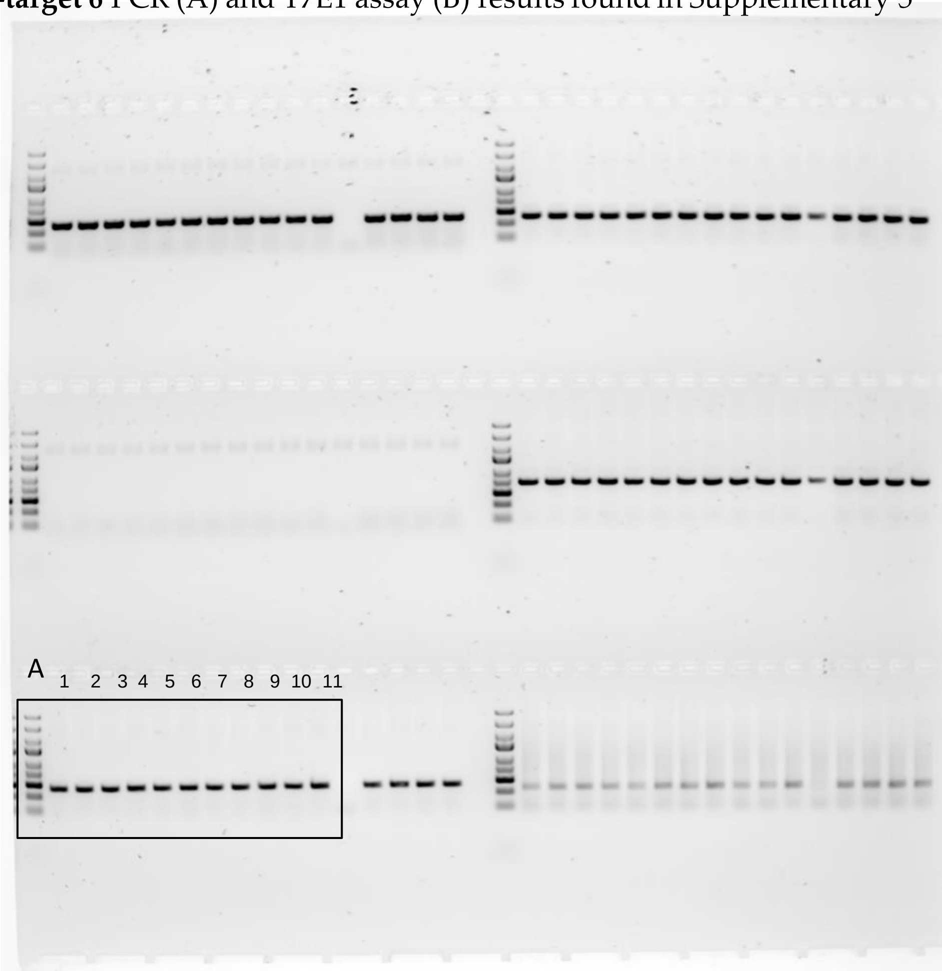

Sample	ID
1	IR64-IRS1421-001-009-005-024
2	IR64-IRS1421-001-009-004-020
3	IR64-IRS1421-026-007-001-017
4	IR64-IRS1421-026-007-001-018
5	IR64-IRS1421-026-007-001-020
6	IR64-IRS1421-026-007-001-021
7	IR64-IRS1421-026-007-002-011
8	IR64-IRS1421-026-007-002-012
9	IR64-IRS1421-026-007-002-024
10	IR64-IRS1421-026-007-003-009
11	IR64-IRS1421-026-007-003-023

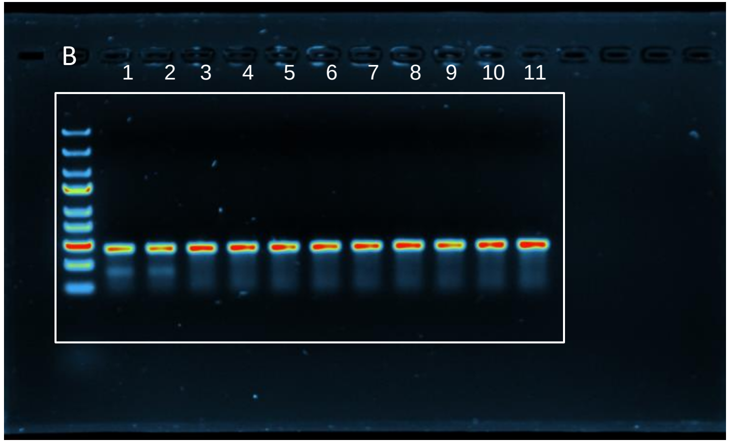

(19) Off-target Screening IR64-IRS1421-T_3 generation

Off-target 7 PCR (A) and T7E1 assay (B) results found in Supplementary 5

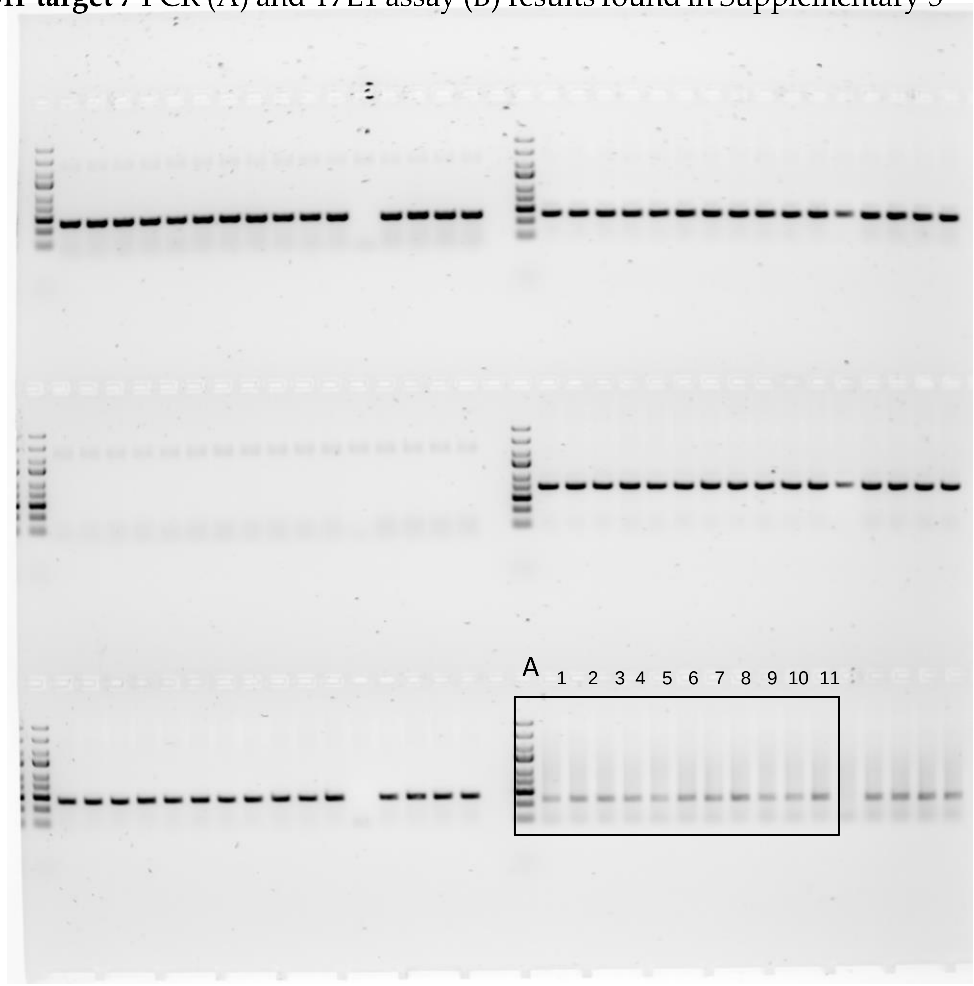

Sample	ID
1	IR64-IRS1421-001-009-005-024
2	IR64-IRS1421-001-009-004-020
3	IR64-IRS1421-026-007-001-017
4	IR64-IRS1421-026-007-001-018
5	IR64-IRS1421-026-007-001-020
6	IR64-IRS1421-026-007-001-021
7	IR64-IRS1421-026-007-002-011
8	IR64-IRS1421-026-007-002-012
9	IR64-IRS1421-026-007-002-024
10	IR64-IRS1421-026-007-003-009
11	IR64-IRS1421-026-007-003-023

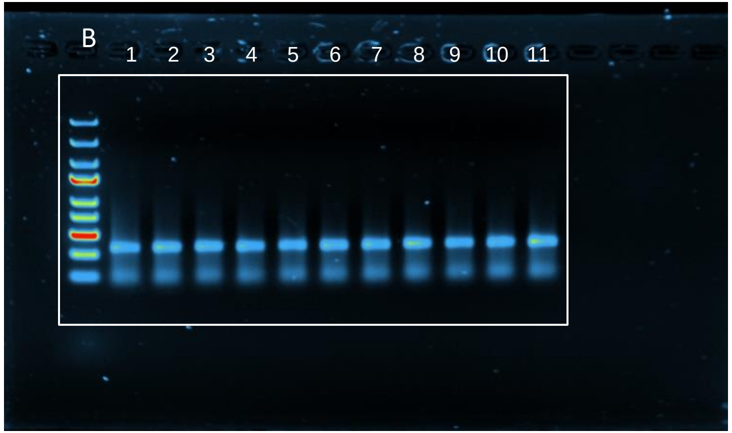

(20) Off-target Screening IR64-IRS1421-T_3 generation

Off-target 8 PCR (A) and T7E1 assay (B) results found in Supplementary 5

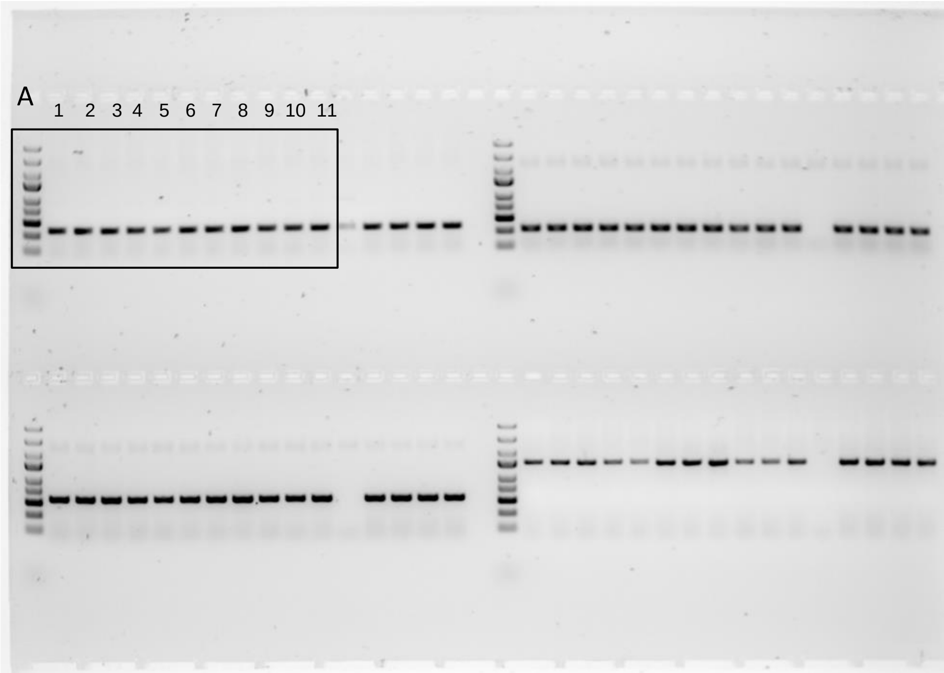

Sample	ID
1	IR64-IRS1421-001-009-005-024
2	IR64-IRS1421-001-009-004-020
3	IR64-IRS1421-026-007-001-017
4	IR64-IRS1421-026-007-001-018
5	IR64-IRS1421-026-007-001-020
6	IR64-IRS1421-026-007-001-021
7	IR64-IRS1421-026-007-002-011
8	IR64-IRS1421-026-007-002-012
9	IR64-IRS1421-026-007-002-024
10	IR64-IRS1421-026-007-003-009
11	IR64-IRS1421-026-007-003-023

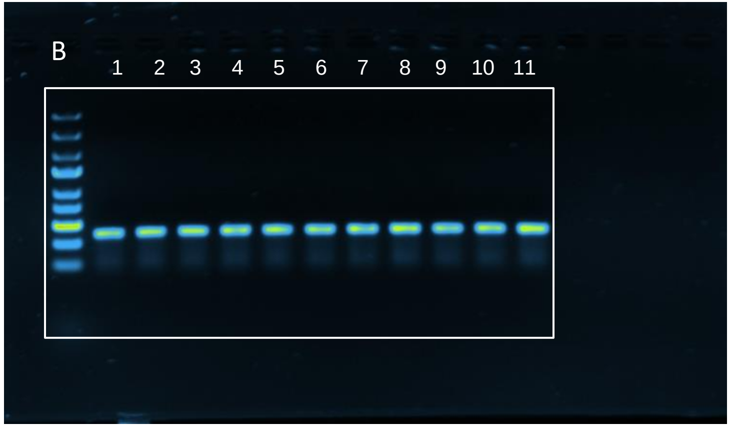

(21) Off-target Screening IR64-IRS1421-T_3 generation

Off-target 9 PCR (A) and T7E1 assay (B) results found in Supplementary 5

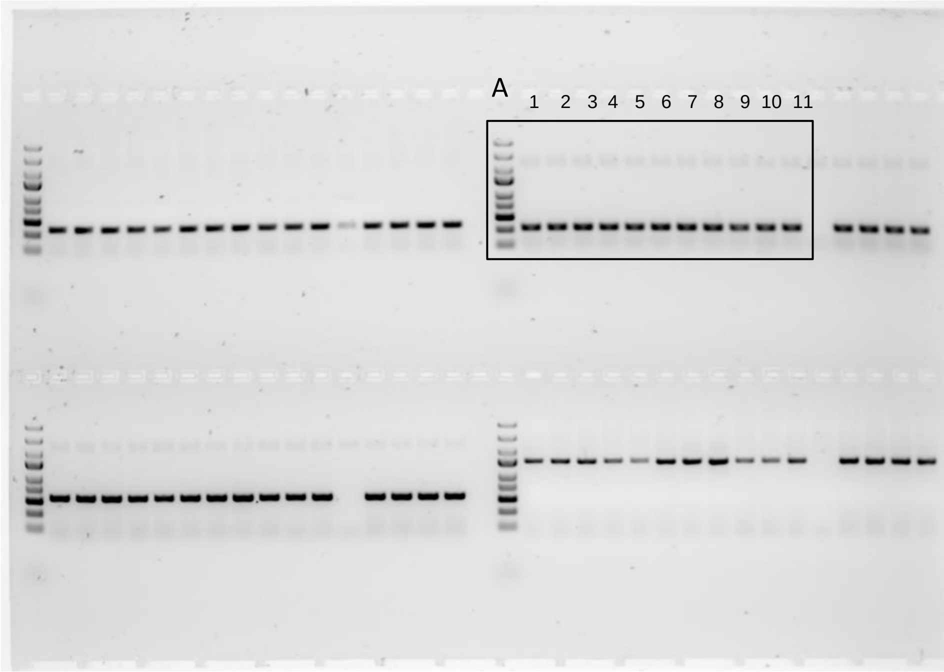

Sample	ID
1	IR64-IRS1421-001-009-005-024
2	IR64-IRS1421-001-009-004-020
3	IR64-IRS1421-026-007-001-017
4	IR64-IRS1421-026-007-001-018
5	IR64-IRS1421-026-007-001-020
6	IR64-IRS1421-026-007-001-021
7	IR64-IRS1421-026-007-002-011
8	IR64-IRS1421-026-007-002-012
9	IR64-IRS1421-026-007-002-024
10	IR64-IRS1421-026-007-003-009
11	IR64-IRS1421-026-007-003-023

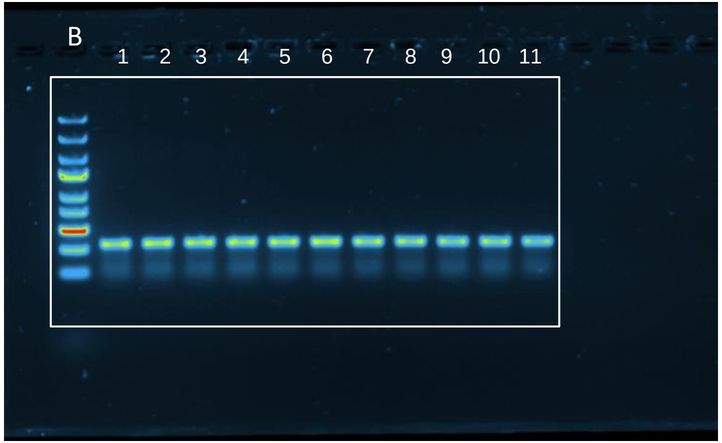

(22) Off-target Screening IR64-IRS1421-T_3 generation

Off-target 10 PCR (A) and T7E1 assay (B) results found in Supplementary 5

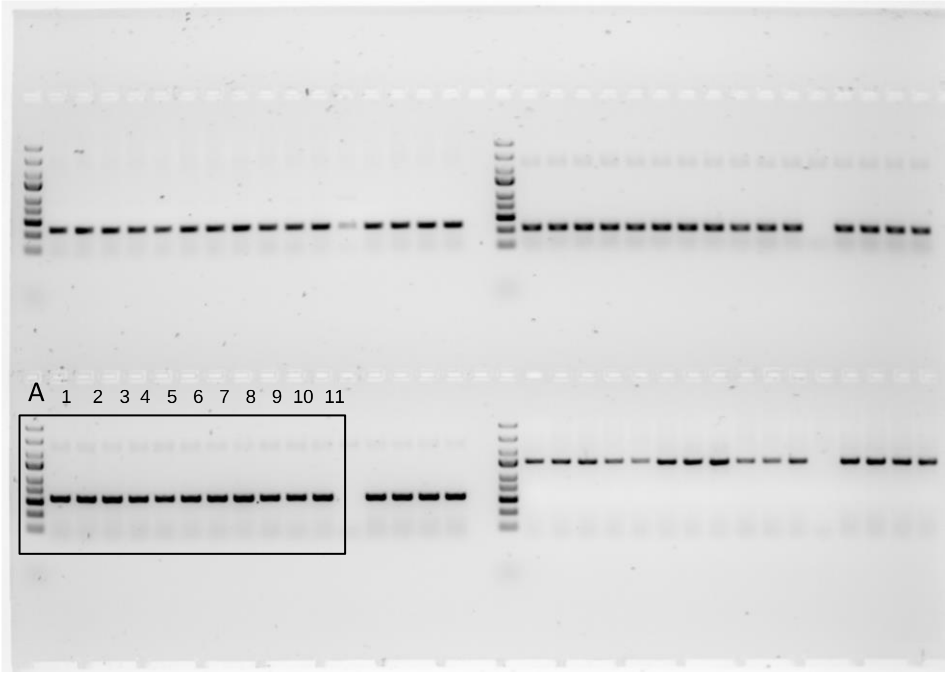

Sample	ID
1	IR64-IRS1421-001-009-005-024
2	IR64-IRS1421-001-009-004-020
3	IR64-IRS1421-026-007-001-017
4	IR64-IRS1421-026-007-001-018
5	IR64-IRS1421-026-007-001-020
6	IR64-IRS1421-026-007-001-021
7	IR64-IRS1421-026-007-002-011
8	IR64-IRS1421-026-007-002-012
9	IR64-IRS1421-026-007-002-024
10	IR64-IRS1421-026-007-003-009
11	IR64-IRS1421-026-007-003-023

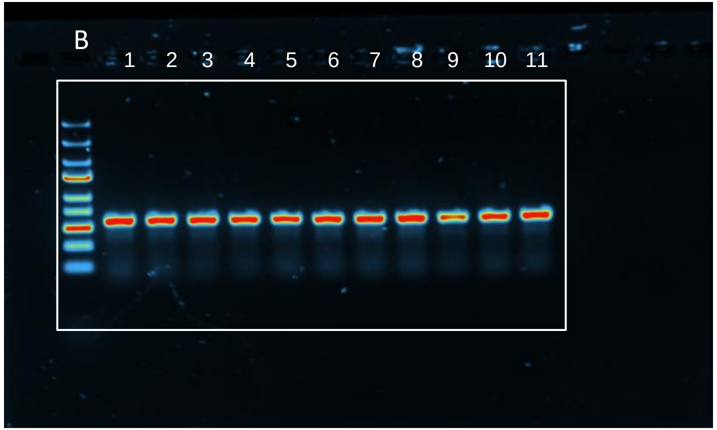

Supplement: Supplementary file 7 [file DataSheet1.PDF]
